# Medullary stromal cells define small intestinal lymph node identity in humans and mice

**DOI:** 10.1016/j.celrep.2025.116441

**Published:** 2025-10-16

**Authors:** Aliia R. Fatkhullina, Johnathan Kent, Hailey Brown, Nathaniel Christiansen, Wioletta Lisicka, Maria Lucia Madariaga, Daria Esterházy

**Affiliations:** 1 Department of Pathology, University of Chicago, Chicago, IL, USA; 2 Committee on Immunology, University of Chicago, Chicago, IL, USA; 3 Department of Surgery, The University of Chicago Medicine, Chicago, IL, USA; 4 Present address: Cardiovascular Institute, Department of Medicine, Perelman School of Medicine, University of Pennsylvania, Philadelphia, PA, USA; 5 Present address: Yale School of Medicine, New Haven, CT, USA

## Abstract

Lymph nodes (LNs) along the murine gastrointestinal tract are immunologically distinct. Here, we investigate whether such an immune dichotomy globally defines gut LN identity and what drives the regional differences. However, we find that, transcriptionally, it is genes associated with stromal cells that define LN location along the intestine, with high conservation between human and mouse. Using LN single-cell RNA sequencing databases and imaging, we pinpoint the underlying reason for our transcriptional signature: a selective enrichment in the small intestine-draining LN medulla for fibroblastic reticular cell subpopulations implicated in extracellular matrix remodeling and stromal cell replenishment, lymphatic endothelial cells, and macrophages. In mice, the unique medullary cellular network is established around weaning age by vitamin A, while the gut microbiota regulates the effect’s amplitude. Our study implicates the LN medulla as contributing to tissue-specific immunity and uncovers privileged access to lipid-soluble vitamins as the pivotal driving force for small intestinal LN architecture.

## In brief

Fatkhullina et al. discover that in humans and mice, the dominant difference between lymph nodes draining the small intestine versus colon and other sites is their stromal cell and macrophage composition. This difference is induced by dietary vitamin A and amplified by the microbiota.

## Graphical Abstract

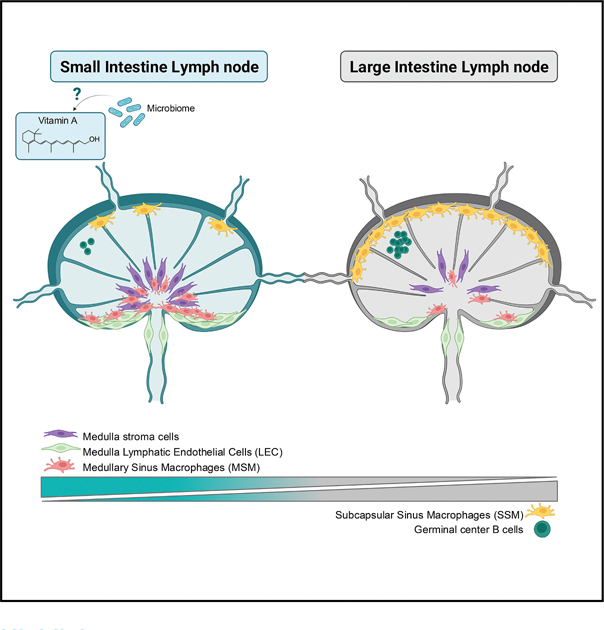

## INTRODUCTION

Lymph nodes (LNs) are secondary lymphoid organs that initiate adaptive immunity in mammals and some birds.^1^ Beyond driving B cell maturation, isotype switching, and T cell fate decisions, they also serve as an innate “firewall” against the spread of local infections. Unlike centralized organs such as the spleen, LNs are strategically distributed to drain specific tissues, enabling localized immune surveillance and tailored responses. In the past decade, we have come to appreciate that in mice, the LNs draining different sites are immunologically distinct^2–4^; however, we know comparatively little about differences between LNs across the human body.^5–7^ That human LNs adapt to the environment they drain is known from studying LNs at a particular location in a healthy versus disease state.^8–10^ Knowing whether all LNs are equal or if they inherently differ in the amplitude and nature of adaptive immune responses they can elicit is important to understanding tissue-specific disease etiologies and devising strategic immunization therapies.

Even less is known about how human LNs vary along the gut, despite more than 150 dedicated to this tissue. In mice, proximal gut-draining LNs (gLNs) are more tolerogenic, whereas distal LNs are more pro-inflammatory, shaped by dendritic cell (DC) profiles, T cell fates, and location-specific fibroblastic reticular cells (FRCs) and lymphatic endothelial cells (LECs).^2,3^ If similar in humans, this could explain why inflammatory bowel diseases arise more frequently in the distal intestine and indicate that tolerance breaks in the proximal LNs in food allergy.

In addition to these gaps of knowledge about the human LNs, previous studies in mice focused mostly on immune cells, leaving the stromal cells (SCs) understudied. The potentially important role of regional SCs is underscored by the fact that neonatal imprinting of gut-draining LNs appears to be sufficient to retain a tolerogenic potential even upon transplantation outside the gut,^11^ and that this potential is dependent on the microbiota and retinoic acid.^12^ It has also recently been reported that mesenteric LNs (mLNs) distinguish themselves from inguinal LNs by an expanded CD34^+^ SC population.^13^ Whether SCs differ between LNs along the gut, and what fundamentally drives regional differences between gLNs, is not fully understood, not even in mice.

We investigated whether LNs along the human gastrointestinal tract differ regionally, how these distinctions compare to mice and what drives them. Combining bulk RNA sequencing (RNA-seq) of LNs and their deconvolution using published single-cell (sc) RNA-seq datasets, along with systematic LN section staining, and environmental and genetic interventions in mice, we uncover that in both species, the strongest regional signatures arise from medullary stromal rather than immune cells.

## RESULTS

### Human mucosal LNs are transcriptionally distinct according to the organ or gut region they drain

**Figure 1.**
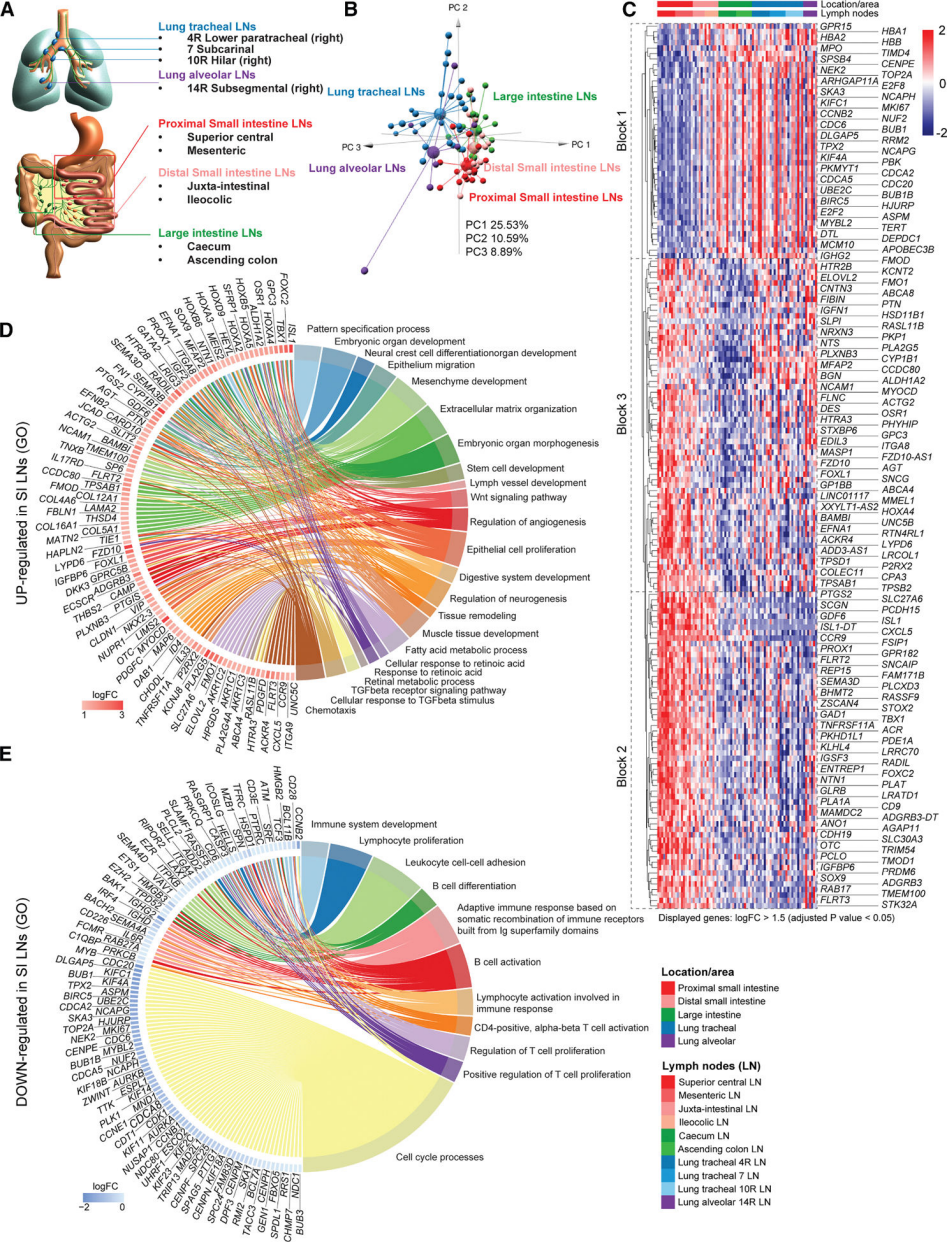
Human mucosal LNs are transcriptionally distinct according to the organ or gut region they drain (A) Schematic of the human lung and intestine. The five anatomical structures and the ten LNs draining them, analyzed by RNA-seq are indicated. (B) Principal-component plot of RNA-seq data of LNs pooled by the anatomical structures they drain. (C) Heatmap and hierarchical clustering of the top DEGs (logFC > 1.5, adjusted *p* value < 0.05) between the superior central LN of the SI versus the cecal LN of the large intestine across all LNs on which RNA-seq was performed. (D and E) Chord diagrams connecting major GOBP pathways and DEGs (adjusted *p* value < 0.05, displayed genes logFC > 1.2) contributing to them that are enriched (D) or de-enriched (adjusted *p* value < 0.05, displayed genes logFC < − 0.4) (E) in the SI LNs. Biological replicates in (B)–(E): superior central LN *n* = 9, mesenteric LN *n* = 9, juxta-intestinal LN *n* = 6, ileocolic LN *n* = 7, cecum LN *n* = 9, ascending colon LN *n* = 8, lung tracheal 4R LN *n* = 9, lung tracheal 7 LN *n* = 8, lung tracheal 10R LN *n* = 9, and lung alveolar 14R LN *n* = 7.

To identify the major transcriptional differences between human intestinal LNs, we performed bulk RNA-seq on whole LNs from organ donors (Figures 1A and S1A). While scRNA-seq and spatial transcriptomics offer higher resolution, bulk RNA-seq avoids issues such as poor SC recovery, cell composition bias, non-representative sections of complex asymmetric tissues, or incomplete transcriptome coverage. We analyzed two representative LN stations from each, the proximal versus distal small intestine and colon (Figure 1A). We also included four lung LN stations from the same donors (Figure 1A) that reflect compartmentalized drainage of another organ and would help us understand which phenomena are unique to the gut. Principal-component analysis (PCA) of the bulk RNA-seq data (Figure 1B; Tables S1A and S1B) revealed clear segregation: small intestinal (SI) LNs from large intestinal (LI) and lung LNs (PC1 and PC2) and lung LNs from gut LNs (PC3). No major differences between alveolar and tracheal LNs were observed (Figure S1D; Table S1M), likely, in part, due to the high variation in the lung LN samples. However, pooled tracheal LNs were transcriptionally distinct from pooled colonic LNs (Figure S1B; Table S1D), and smoking status also impacted the tracheal LN expression profile (Figure S1C; Table S1E), indicating that lung LNs are, in principle, affected by the environment they drain. By contrast, intestinal LNs show strong segment-specific profiles, whereby the genes most differentially expressed between the superior central (SI) and caecal LN (LI) show an intermediate expression level in the distal small intestine (Figure 1C). The 2,170 differentially expressed genes (DEGs) (Table S1C) represented more upregulated genes (1,495) than downregulated genes (675) in the SI versus LI LNs, which was also true for transcripts enriched by more than a log fold change (logFC) of 1.5 (120 in superior central versus 45 in cecal LN). Interestingly, the genes upregulated in the LI LNs displayed similar expression patterns in the lung LNs (Figure 1C, Block 1); the transcripts enriched in the SI LNs fell into two major blocks, one where, again, the lung LNs behaved like the LI LNs (Figure 1C, Block 2) and one where it resembled the distal SI (Figure 1C, Block 3). Overall, this heatmap matched the PCA, whereby lung LNs were more like colonic LNs. We next wondered what biological processes these genes were involved in. Gene ontology (GO) analysis yielded that transcripts enriched in the SI LNs (superior central versus caecal LNs) fell into categories related to embryonic development, vascular development (including lymph vessel development), Wnt signaling, tissue remodeling, and retinol/lipid/fatty acid metabolism (Figure 1D; Table S1F). Genes downregulated in SI LNs and consequently enriched in LI LNs were primarily involved in T and B cell activation, differentiation, and proliferation, with genes participating in the cell cycle being particularly prominent (Figure 1E; Table S1G).

These data demonstrate that human LNs are distinct in dependence on the organ they drain and, within the gut, the particular intestinal segment.

### Human SI LN-enriched transcripts primarily map to medullary sinus stromal cells, while those of LI LNs map to germinal center B cells

**Figure 2.**
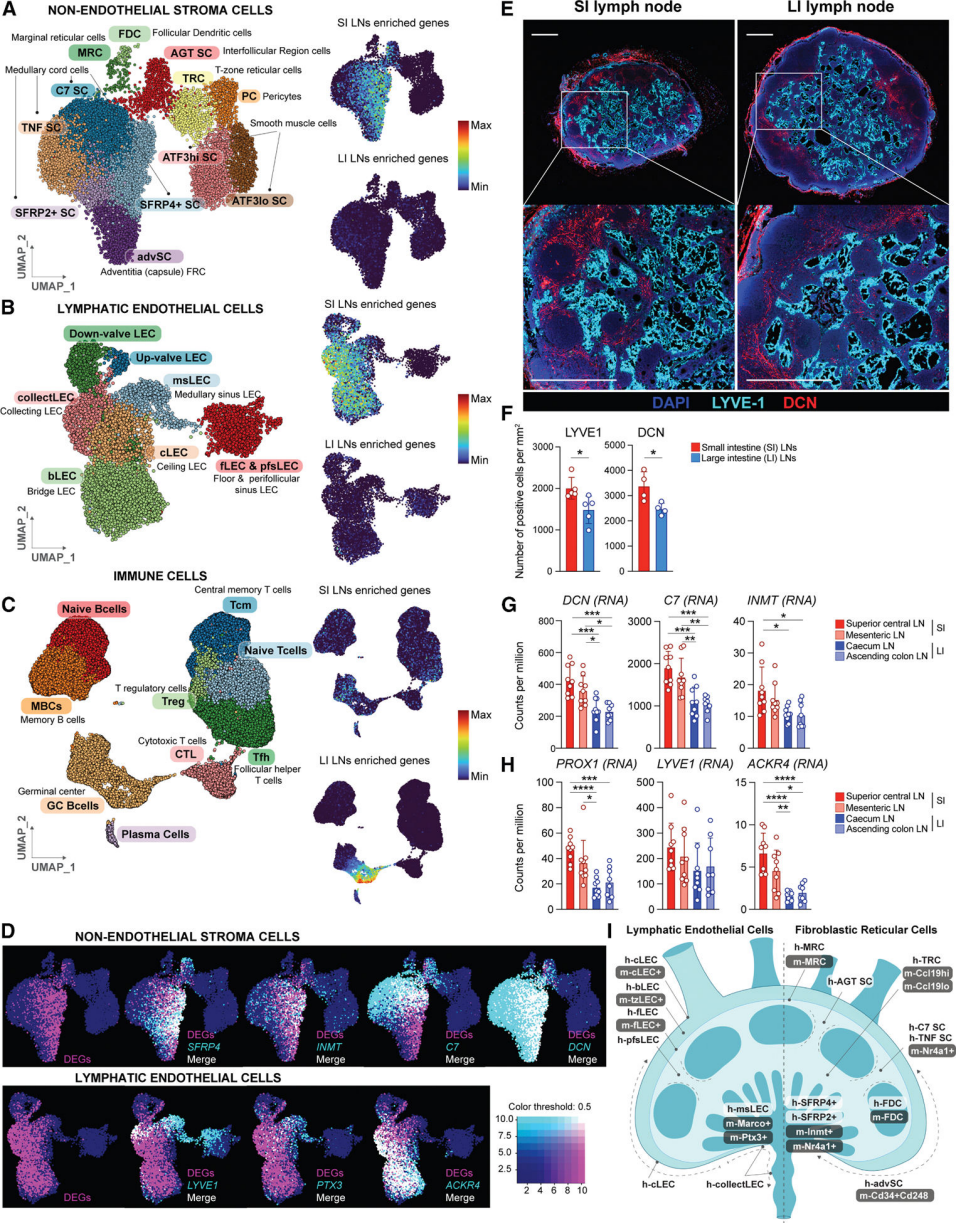
Human SI LN enriched transcripts primarily map to medullary sinus SCs, while those of LI LNs map to GC B cells (A–C) UMAP plots of NESC types in human LNs based on Abe et al.^6^ (A), LEC types based on Takeda et al.^7^ (B), or immune cell types, based on De Martin et al.^20^ (C), colored by cell type (left) or by intensity (on min-max scale) of representation of DEGs enriched in the SI LNs (upper right) or LI LNs (lower right). (D) UMAP plots of NESC (top) and LECs (bottom) are colored according to the expression of DEGs (magenta) and indicated marker gene (cyan), whereby coinciding expression is marked as white. (E) Representative cross-section of human SI (superior central) and LI LN (caecal) stained immunofluorescently with DAPI (dark blue), anti-LYVE-1 (cyan), and anti-DCN (red). Scale bars: 400 μm (top) and 800 μm (bottom). Average dimensions of superior central LN: 2.5 × 3.5 × 3 mm^3^ and of cecal LN: 5 × 5 × 4 mm^3^. (F) Quantification of LYVE-1 (*n* = 5)- and DCN (*n* = 4)-positive cells per mm^2^ in cross-sections of superior central (SI) or cecal (LI) LNs. (G and H) Normalized CPM of FRC transcripts (G) or LEC transcripts (H) from indicated LNs as determined by RNA-seq. Superior central LN *n* = 9, mesenteric LN *n* = 9, cecal LN *n* = 9, and ascending colon LN *n* = 8. (I) Schematic of LN, with LEC subtypes, indicated on the left and non-endothelial SC/FRC subtypes on the right. Human nomenclature is highlighted in white and mouse nomenclature in gray. Data are represented as mean ± SEM, *p < 0.05, **p < 0.01, ***p < 0.001, and ****p < 0.0001 by paired *t* test.

To identify the cell types driving location-specific signatures, we queried the top 200 SI and LI LN enriched DEGs against the Immgen/My Geneset database (Figures S2A and S2B). SI LN-enriched transcripts were attributed mainly to stem cells, macrophages, granulocytes, monocytes, and SCs (underrepresented in Immgen), while LI LN-enriched transcripts were also associated with B and T cells. Previous work using flow cytometry, CyTOF,^14–16^ and scRNA-seq^17,18^ has profiled LN immune cells, and scRNA-seq has further revealed stromal heterogeneity,^6,7,19^ with LEC, blood endothelial cell (BEC), and FRC subtypes mapping to distinct LN regions (sinuses, T and B cell zones, interfollicular regions, and medulla). We, therefore, next used published scRNA-seq datasets of LN SCs^6^ or including adaptive immune cells^20^ to refine the gene-to-cell type assignment. Plotting our DEGs onto a uniform manifold approximation and projection (UMAP) plot of non-endothelial SCs (NESCs) (Figures 2A–2D and 2I) showed SI LN-enriched transcripts localized to the medulla and interfollicular region, including *SFRP4*+ cells, adventitia (adv)SCs, and parts of *C7*+ and *SFRP2*+ clusters, while LI LN-enriched transcripts were not associated with NESCs. Similarly, SI LN-enriched transcripts were mostly assigned to LECs (Figure 2B), especially bridge LECs (bLECs) and collecting vessel LECs (collectLECs), which are both *ACKR4*+ (Figures 2B and 2D). However, all LEC subtypes, except floor LECs (fLECs) and perifollicular sinus LECs (pfsLECs), displayed partial overlap with SI LN-enriched transcripts, including medullary sinus (ms) LECs (*PTX3*+, Figure 2D). By contrast, SI LN-enriched transcripts were not strongly expressed in T or B cells (Figure 2C), whereas LI LN-enriched transcripts clustered in germinal center (GC) B cells, explaining the cell cycle gene enrichment (Figure 1E). Across the scRNA-seq datasets, most SI LN-enriched transcripts were attributed to non-hematopoietic cells: ∼one-third FRCs, ∼half LECs, and ∼15% BECs (Figure S2C). LI LN-enriched transcripts were mainly immune-derived, with ∼two-third GC B cells, whose proportion was higher in LI LNs (Figures S2D and S2E). Neither dataset scRNA-seq included innate immune cells, but the anatomical locations of the SCs to which we attributed our genes to^6,7,19^ indicated that most SI LN-enriched transcripts are expressed by medullary and medullary sinus SCs (Figure 2I; Table S2A). Focusing on SI LN-defining genes, we asked whether their enrichment reflected more numerous medullary SCs in SI versus LI LNs, higher per-cell gene expression, or both. Sections of superior central SI and cecal LI LNs were stained for LYVE1 (medullary LECs) and Decorin (DCN)—a marker for medullary advSCs, *SFRP4*+, *SFRP2*+, *C7*+, and *TNF+* SCs^6^ (all FRCs of our DEGs colocalized with this marker, Figure 2D)—and the stain-positive cells were quantified (Figure 2E and 2F). SI LNs harbored more LYVE1+ and DCN+ cells, and analyzing the counts per million (CPM) of further genes indicative of FRC and LEC genes showed proximal-to-distal gradients (Figures 2G and 2H). To answer whether our DEGs were exclusively accounted for by increased cell numbers in the SI compared to LI LNs, we normalized the CPM of our whole LN RNA-seq to the CPM of *DCN* (NESCs) or *PROX1* (lineage-defining LEC transcription factor). Normalizing DEGs to *DCN* or *PROX1* indicated that the FRC gene enrichment resulted from both higher cell numbers and per-cell expression, while LEC gene expression differences were mainly due to more LECs present in the SI LNs (Figures S2F and S2G; Tables S1H and S1I). This explained our GO results: lymphangiogenic pathways reflected a denser lymphatic vasculature, developmental pathways indicated a putative FRC stem cell-like function of some medullary SCs and were likely also enriched due to increased cell numbers, and metabolic pathways reflected regional lipid-rich lymph imprinting.

Together, these analyses reveal a unique expansion of medullary LEC and FRC subsets in SI LNs, distinguishing them from LI and lung LNs.

### Mouse SI LNs are transcriptionally distinct from those draining colon and skin, and the difference is dampened but not lost in the absence of a microbiota

**Figure 3.**
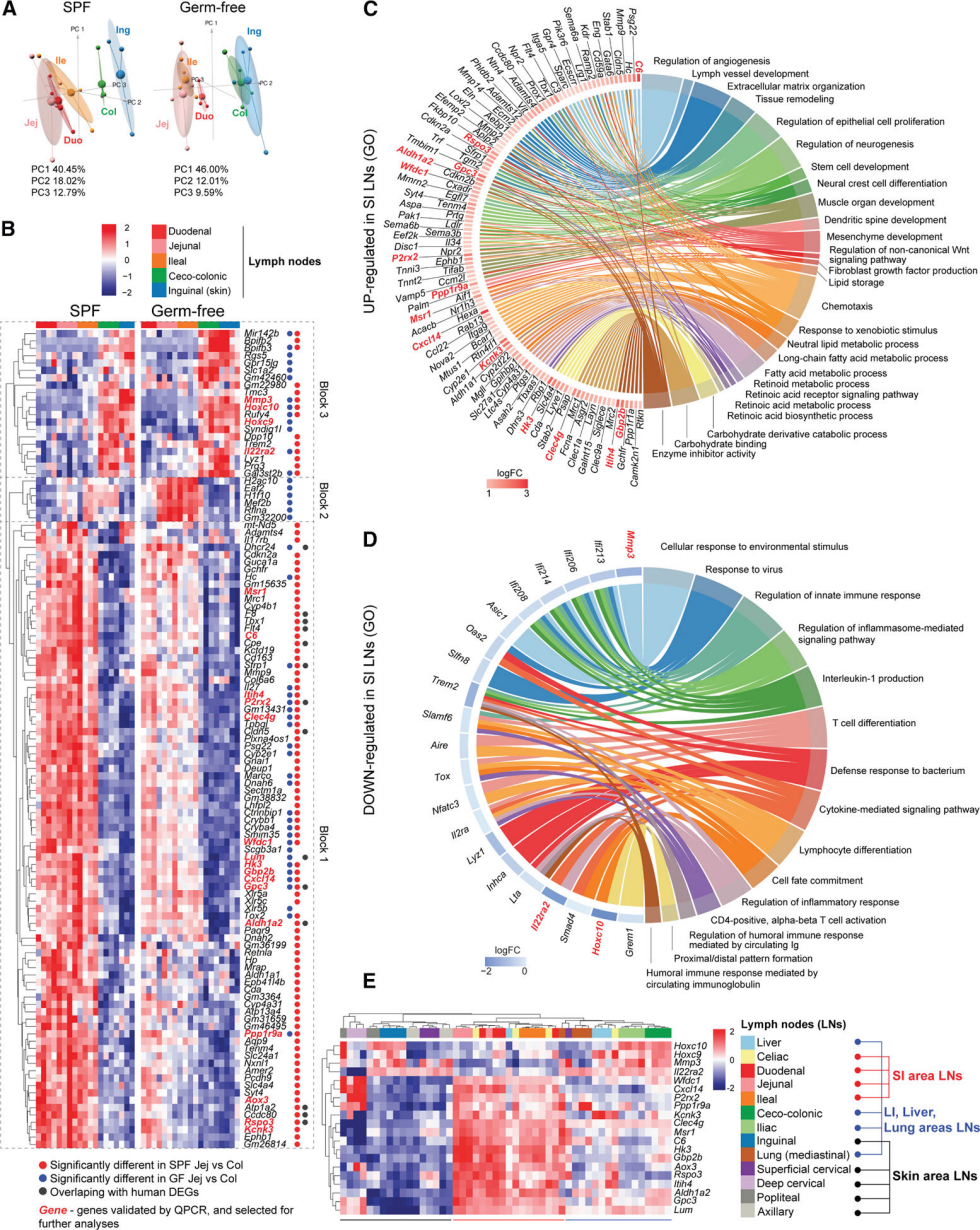
The mouse SI LNs are transcriptionally distinct from those draining colon and skin, and the difference is dampened but not lost in the absence of microbiota (A) Principal-component plots of RNA-seq data of LNs from SPF (left) or GF (right) mice. Duo, duodenal; Jej, jejunal; Ile, Ileal; Col, ceco-colonic; and Ing, inguinal LN. (B) Heatmap and hierarchical clustering of the top DEGs (logFC >1.5) between jejunal and ceco-colonic LN of SPF and GF mice across all LNs on which RNA-seq was performed. (C and D) Chord diagrams connecting major GOBPs and DEGs (adjusted *p* value < 0.05) contributing to them that are enriched (C) or de-enriched (D) in the SI LNs of SPF mice. Symbols of transcripts subsequently measured by qPCR are in red. Biological replicates in (A)–(D): SPF: duo LN *n* = 4, jej LN *n* = 4, ile LN *n* = 4, col LN *n* = 4, ing LN *n* = 3; GF: duo LN *n* = 3, jej LN *n* = 4, ile LN *n* = 4, col LN *n* = 4, and ing LN *n* = 4. (E) Heatmap and hierarchical clustering of gene expression of indicated transcripts in indicated LNs measured by qPCR. *n* = 4 except for popliteal and deep cervical LNs (*n* = 3 each).

Bulk whole LN RNA-seq uncovered regional differences in human gut LNs not previously noted in mice.^2,3^ Since the mouse model is central to mucosal immunology and gastrointestinal disease modeling, we asked whether increased medullary stromal cellularity in SI LNs is conserved or if other signatures dominate. Leveraging the genetic and environmental control when using mice, we performed bulk RNA-seq on murine gLNs from germ-free (GF) and specific pathogen-free (SPF) C57BL/6 mice, with inguinal LNs as non-gut controls. PCA showed that SI LNs clustered apart from LI and inguinal LNs in both SPF and GF mice. In SPF mice, duodenal and jejunal LNs clustered most closely among SI LNs, with LI LNs remaining distinct from inguinal LNs. In GF mice, jejunal and ileal LNs were more similar, while LI and inguinal LNs no longer separated (Figure 3A; Tables S3A and S3B). Of the 609 (SPF) and 161 (GF) DEGs between jejunal and ceco-colonic LNs, more were enriched in SI than LI LNs (454 versus 155; 119 versus 42), with many highly upregulated (logFC > 1.5) (Table S3C). Heatmaps revealed three gene groups: SI LN-enriched transcripts largely preserved in GF (block 1), LI LN-enriched transcripts consistent across SPF/GF (block 3), and a subset shifting from LI LN-enriched (SPF) to SI LN-enriched (GF) (Figure 3B, block 2). GO analysis showed a strong resemblance to human SI-versus LI LN-enriched pathways (Figures 3C and 3D; Tables S3E and S3F): SI LN-enriched transcripts were linked to vasculature/lymph vessel development, other developmental processes, and lipid/retinoic acid metabolism, while LI LN-enriched transcripts related to immune activation, lymphocyte differentiation, and cell cycle. SPF versus GF differences were notable mainly in jejunal LNs, affecting angiogenesis, tissue remodeling, and lipid metabolism (Table S3D; Figures S3B and S3C; Tables S3G and S3H), consistent with microbiota-driven stromal imprinting.^13^ Overall, the microbiota influenced SI LNs more than LI LNs but did not drive the SI-LI LN dichotomy, which remained largely preserved. With access to mouse LNs across the body, we tested whether the SI versus LI LN gene signatures were unique to these LNs. We chose a panel of genes whose transcripts displayed at least a logFC of >1.5 between jejunal and ceco-colonic LNs from SPF or GF mice and were not B cell-associated (Figures 3C and 3D, red, bold) and quantified their expression in LNs from different areas by qPCR. This analysis revealed three key points (Figure 3E): SI LN signature transcripts were uniquely enriched in SI LNs (Figure 3C) and de-enriched in LI LNs (Figure 3D); LI LNs partially resembled SI LNs for SI-enriched transcripts, reflecting the gut environment; and unsupervised clustering showed skin-draining LNs grouped together, SI LNs as a separate cluster, and LI LNs grouped with liver and lung LNs.

In sum, murine SI LNs are distinguished from LI and other organ-draining LNs by pathway enrichments similar to human SI LNs, a pattern maintained without microbiota.

### Murine SI LN-enriched transcripts primarily map to lymph node sinus cells, while those of LI LNs map to B cells

**Figure 4.**
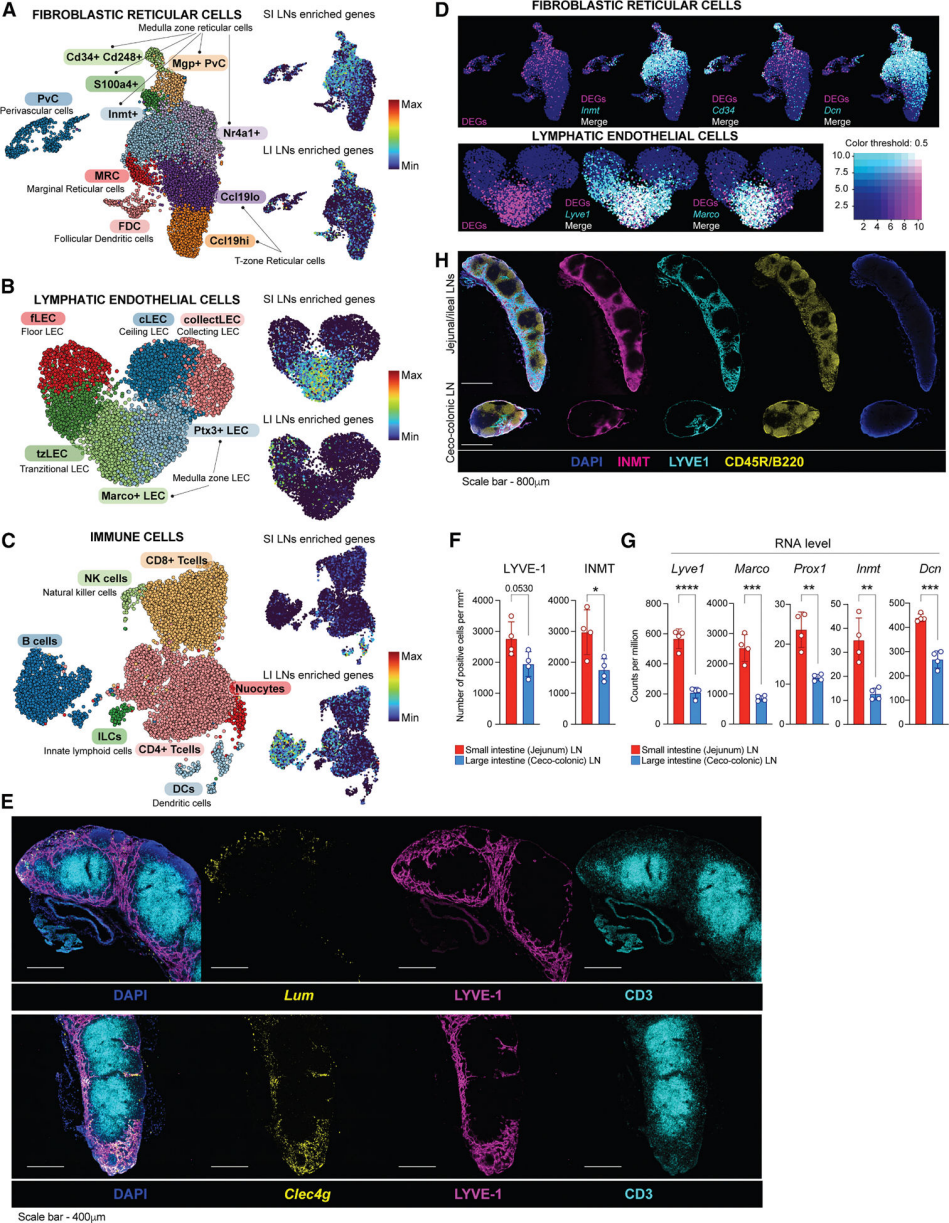
Murine SI LN-enriched transcripts primarily map to lymph node sinus cells, while those of LI LNs to B cells (A–C) UMAP plots of non-endothelial SC types in mouse LNs of (A) and lymphatic endothelial cell types based on Lenti et al.^23^ (B), or immune cell types based on Tong and Mang et al.^18^ (C) colored by cell type (left) or by intensity (on min-max scale) of representation of DEGs enriched in the SI LNs (upper right) or LI LNs (lower right). (D) UMAP plots of non-endothelial stroma cells (top) and LECs (bottom), colored according to the expression of DEGs (magenta) and indicated marker gene (cyan), whereby coinciding expression is marked as white. (E) Representative cross-section of murine jejunal LNs stained immunofluorescently with DAPI (dark blue), anti-CD3 (cyan), anti-LYVE1, and RNA probes against *Lum* or *Clec4g* (yellow). Scale bars, 400 μm. (F) Quantification of LYVE-1 (*n* = 4)- and INMT (*n* = 4)-positive cells per mm^2^ in the cross-sections of jejunal (SI) or ceco-colonic (LI) LNs. (G) Normalized CPM of indicated transcripts and LNs as determined by RNA-seq. (H) Representative cross-section of murine small intestine (mesenteric) LN chain and ceco-colonic LN-stained immunofluorescently with DAPI (dark blue), anti-B220 (cyan), anti-INMT (magenta), and anti-LYVE1 (green). Scale bars, 800 μm. Data are represented as mean ± SEM, *p < 0.05, **p < 0.01, ***p < 0.001, and ****p < 0.0001 by two-tailed Student’s *t* test.

To identify cell types driving the SI versus LI LN signatures, we analyzed our DEGs in the mouse Immgen RNA-seq database (Figures S4A and S4B; Tables S4A and S4B). SI LN-enriched transcripts were mainly expressed by monocytes, macrophages, and SCs, while LI LN-enriched transcripts were assigned to B cells as well as SCs. To further confirm that our DEGs came mainly from SCs, we performed RNA-seq on gut and inguinal LNs from *Rag1* knockout (*Rag1*^−/−^) mice, whose LNs are de-enriched for T and B cells, two major contributors to LN cellularity. PCA showed that *Rag1*^−/−^ LNs clustered similarly to SPF mice, but with greater separation (Figure S4C). DEG analysis between jejunal and colonic LNs revealed 2,948 genes, including all SI LN-enriched transcripts from SPF and GF mice plus additional genes (Figures S4D and S4E; Table S4C). Interestingly, LI LN-enriched transcripts overlapped between SPF/GF and *Rag1*^−/−^ mice (e.g., *Il22r2a*, *Hoxc9*, *Hoxc10*, and *Mmp3*), but, evidently, key B cell proliferation genes enriched in SPF LI LNs (*Nuggc*, *Mybl1*, *Aicda*, and *Mef2b*) were missing, while additional genes appeared in *Rag1*^−/−^ LI LNs (Figure S4F). Thus, the findings in this mouse model are in line with the observation that SI LN identity is not primarily driven by adaptive immune cells and unveil that LI LNs also have a non-adaptive immune cell signature that is more evident when B cells are absent. To obtain higher-resolution insights into the cell types responsible for murine DEGs between LNs, we again consulted previously published scRNA-seq datasets of LN SCs and immune cells.^18^ Focusing on FRC subpopulations, SI LN-enriched transcripts were predominantly attributed to *Cd34* +*Cd248*+, *S100a4*+, *Mgp*+PvC, *Inmt*+, and *Nr4a1*+ cells on a UMAP (Figures 4A and D), all located in the medulla, adventitia, or interfollicular space (Figure 2I). All are also *Dcn*+, analogous to human SI LN FRCs (Table S2A). LI LN-enriched transcripts showed some enrichment in follicular dendritic cells (FDCs) and *Cd34*+*Cd248*+ cells. Among LECs, SI LN-enriched transcripts colocalized with *Marco*+ and *Ptx3*+ LECs, which also sit in the medulla (Figures 4B and 4D), while LI LN-enriched transcripts lacked LEC signatures. Also, similar to our finding in humans, medulla LECs were enriched for *Lyve1* (Figure 4D). SI LN-enriched transcripts were also associated with DCs, whereas LI LN-enriched transcripts originated mainly from B and T cells, consistent with GO analysis (Figures 4C and 3D). Indeed, LI LNs contained more GC B cells by flow cytometry (Figure S4G). Using SC scRNA-seq and Immgen data, we attributed the DEGs we had monitored across LNs in the body (Figure 3E) to cell types: *Lum*, *Gpc3*, *Cxcl14*, and *Mmp3* to *Inmt*+, *Nr4a1*+, *Il6*+, and *Cd34* +*Cd248*+ cells; *Rspo3* to *Inmt*+ cells and MRCs; *Aox3* to *Inmt*+, *Ccl19*hi, and *Cd34*+*Cd248*+ cells; *Kcnk3* to Inmt+ cells; *Aldh1a2* to *Cd34*+ cells; *Msr1* to fLECs and msLECs; *Clec4g* and *P2rx2* to msLECs; and *Hoxc9*, *Hoxc10*, *Itih4*, and *Ppp1r9a* were lowly expressed in all FRCs (Tables S2A and S2B). By Immgen and literature,^21,22^ *Aldh1a2* was assigned to DCs and Thetis cells, *Il22ra2* to DCs, and *Hk3*, *C6*, and *Gbp2b* to macrophages (Tables S2A and S2B). We validated the localization of *Lum* and *Clec4g* to the medulla (marked by a denser lymphatic network) by RNAscope (Figure 4E) and of LYVE1+ and INMT+ cells by antibody staining (Figure 4H). To again test if SI LN transcript enrichment reflected increased cell numbers or per-cell expression, we quantified LYVE1+ and INMT+ cells in jejunal versus ceco-colonic LNs. SI LNs contained more of these cells (Figure 4F), correlating with lower CPM for several LEC and medullary SC genes (Figure 4G). Normalization to *Prox1* or *Inmt* reduced but did not fully eliminate the SI versus LI differences (Table S3I).

Overall, these data reveal that in mice, like humans, medullary FRCs/LECs and GC B cells are enriched populations in SI LNs and LI LNs, respectively.

### Murine medullary sinus macrophages are more abundant in SI compared to colonic LNs and transcriptionally distinct

**Figure 5.**
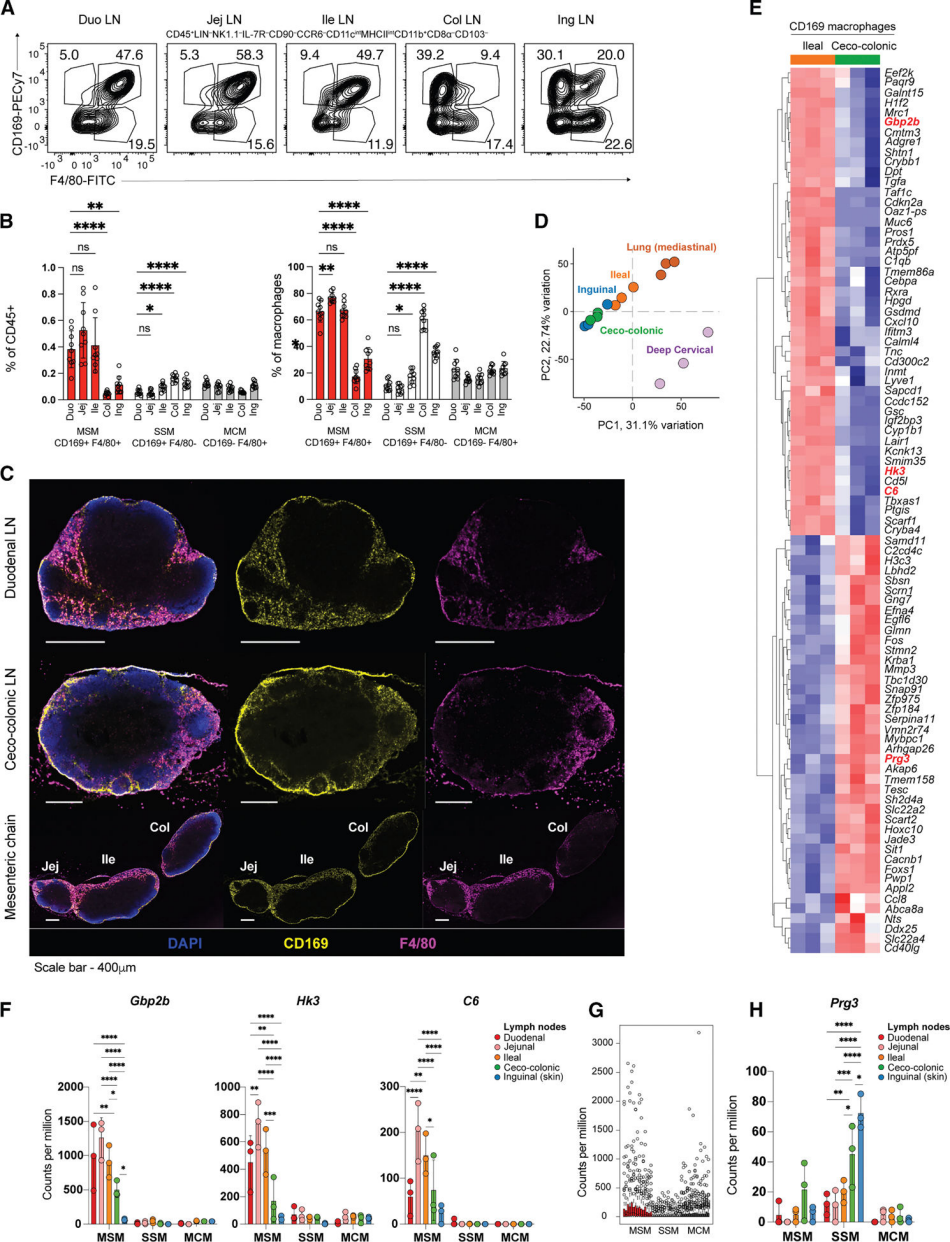
Murine medullary sinus macrophages are more abundant in SI than colonic LNs and transcriptionally distinct (A and B) Representative flow cytometry plots (A) and average percentages (B) of LN macrophage subpopulations in duodenal (duo), jejunal (jej), ileal (ile), ceco-colonic (col), and inguinal (ing) LNs of 7-week-old C57BL/6 mice out of CD45^+^ (left) or total macrophages (right) (*n* = 8). Data were pooled from two independent experiments. (C) Representative cross-section of murine duodenal LN (top), ceco-colonic LN (center), or mesenteric LN chain stained immunofluorescently with DAPI (dark blue), anti-CD169 (yellow), and anti-F4/80 (magenta). Scale bars, 400 μm. (D) Principal-component plot of RNA-seq data of actively translated mRNA pull-down from CD169^+^ cells in the indicated LNs (*n* = 3 per LN type). (E) Heatmap and hierarchical clustering of DEGs (logFC > 1.5) between CD169^+^ cells from ileal and ceco-colonic LNs as determined by RNA-seq of actively translated mRNA. Symbols of transcripts measured by qPCR or pursued in bulk-sorted macrophage subsets (E) are in red. (F) Normalized CPM of indicated macrophage transcripts in sorted MSM, SSM, and MCM from the indicated LNs (*n* = 3). (G) Normalized CPM of all DEGs between ileal and ceco-colonic LNs in sorted MSM, SSM, and MCM (left to right per cell type duo, jej, ile, col, and ing) as determined by RNA-seq. (H) Normalized CPM of the indicated macrophage gene in sorted MSM, SSM, and MCM from the indicated LNs (*n* = 3). MSM, medullary sinus macrophage; SSM, subcapsular sinus macrophage; and MCM, medullary chord macrophage. Data are represented as mean ± SEM; ns, not significant; *p < 0.05, **p < 0.01, ***p < 0.001, and ****p < 0.0001 by ANOVA.

Beyond SCs and B cells, macrophages and DCs were also populations associated with DEGs between SI and LI LNs. Differences in DCs between gLNs are well characterized,^2,3^ but not macrophages. Among the human DEGs linked to macrophages were *CFH*, *PDGFC*, *TNFRSF11*, and *HTRA3*, which showed a proximal-to-distal gradient in CPM (Figure S5A). In mice, LN macrophage profiles could be examined in more depth due to the easier access to materials and genetic tools. LN macrophages occur as three major subtypes^24^: subcapsular sinus macrophages (SSMs), medullary sinus macrophages (MSMs), and medullary cord macrophages (MCMs), distinguished in mice as CD169^+^F4/80^–^, CD169^+^F4/80^+^, and CD169^–^F4/80^+^, respectively. We first assessed whether their distribution was consistent across gLNs and compared this to inguinal LNs by flow cytometry. We found that SI LNs were dominated by MSMs, while LI LNs by SSMs, resembling inguinal LNs (Figures 5A, 5B, and S5B). MCM frequencies remained unchanged, and this flipped distribution was also visible on LN sections (Figure 5C). Since the transcripts of genes putatively expressed by macrophages (e.g., *C6*, *Hk3*, and *Gbp2b*) were enriched in SI, we predicted these were preferentially expressed by MSMs. To test this, we devised a two-pronged strategy: first, bulk RNA-seq of SSMs, MSMs, and MCMs sorted from gLNs and inguinal LNs (Tables S5A and S5B). Despite stringent gating, this approach showed the expected variable contamination from adaptive immune cell RNA, due to natural killer, B, and T cells acquiring macrophage membrane blebs during LN digestion.^25^ Our second strategy was to pull down actively translating ribosomes from CD169^+^ cells by crossing CD169-Cre with Ribotag mice^26^ (Figures S5C and S5D). This sacrificed the resolution of SSMs versus MSMs but increased the specificity to genes expressed by macrophages. While the prolonged pull-down incubation led to RNA degradation in chylous lymph-rich upper SI LNs, ileal LN CD169^+^ RNA was of good quality and could be compared to LI, mediastinal, deep cervical, and inguinal LNs (Tables S5C and S5D). PCA showed that macrophages in ileal versus ceco-colonic LNs segregated (Figure 5D), with ceco-colonic LNs most similar to inguinal, while macrophages in deep cervical and mediastinal LNs were most distinct. Among the DEGs between ileal and ceco-colonic CD169^+^ cells (Figure 5E; Table S5E), and several transcripts previously identified as SI LN-enriched, *Hk3*, *C6*, and *Gbp2b* were again enriched in SI LN CD169^+^ cells. To assign DEGs to MSMs versus SSMs (both CD169^+^), we leveraged our bulk RNA-seq dataset, as background T and B cell mRNA does not interfere when testing a predefined gene list. Most DEGs were enriched in MSMs, followed by MCMs (Figure 5G). Further, *Hk3*, *C6*, and *Gbp2b* were not only MSM-specific but also displayed a proximal-to-distal gradient (Figure 5F), with *Hk3* mRNA localizing to the medullary sinus (Figure S5E). By contrast, *Prg3*, enriched in ceco-colonic CD169^+^ cells, was preferentially expressed by SSMs (Figure 5H).

These analyses show that LN macrophage subsets also vary by LN identity. SI LNs are particularly enriched in MSMs, matching the LN region where SCs are most abundant.

### SI LN-enriched cellular and transcriptional signatures are conserved between mouse and human

**Figure 6.**
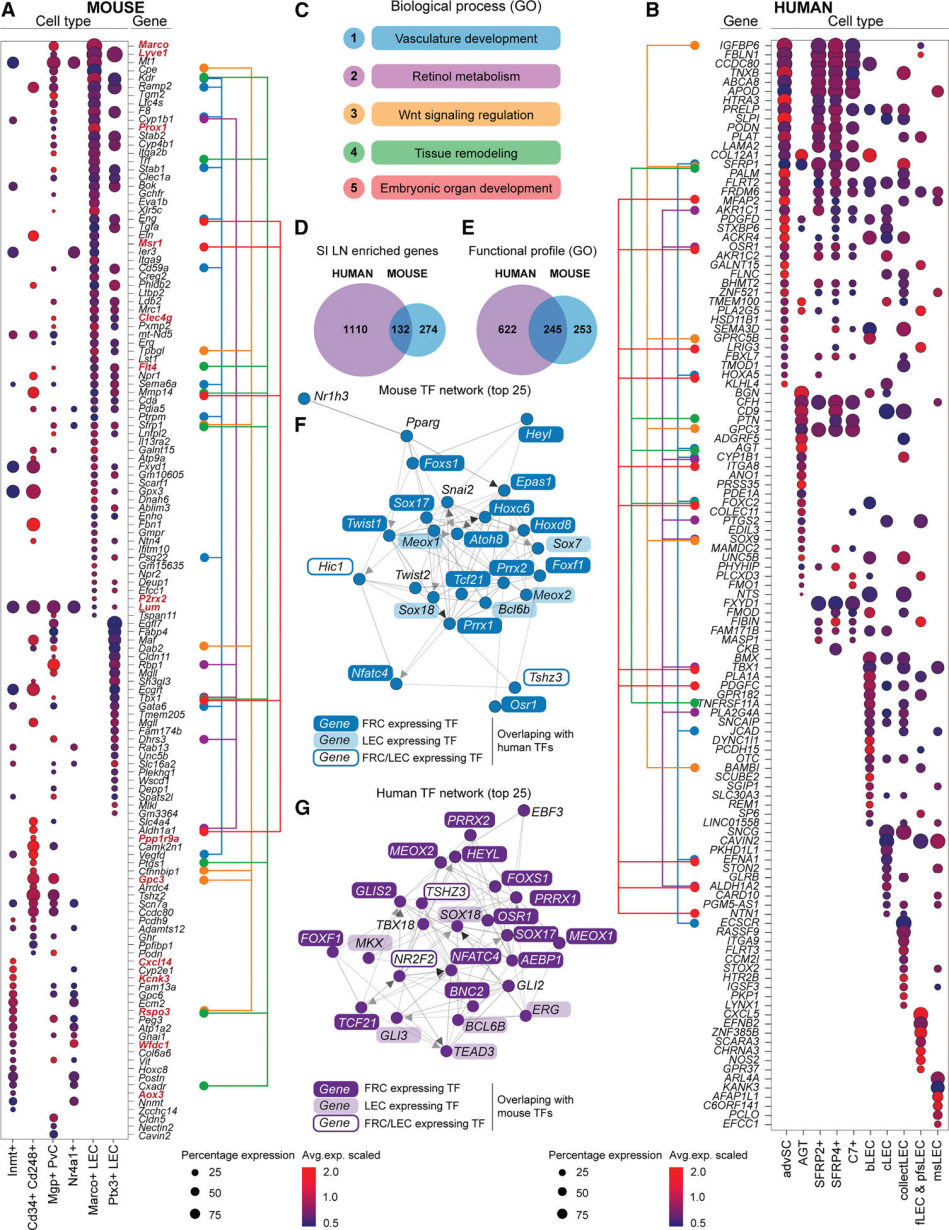
SI LN-enriched cellular and transcriptional signatures are conserved between mouse and human (A–C) Dot plots of the top 150 DEGs between small and large intestine draining LNs and their expression level and percentage of expression in the major murine (A) or human (B) SC types to which DEGs primarily were attributed. The major biological processes (C) to which they belong are color-coded by process in the subway map to the right (A) or left (B) or dot plot. (D and E) Overlap of transcripts (D) and GOBPs (E) between human and mouse of transcripts enriched in the SI LNs of each species (comparisons from Figures 1C and 3B SPF mice). (F and G) Predicted transcription factor network underlying DEGs between SI and LI LNs in mice (F) and humans (G). TF transcripts are highlighted according to whether they regulate FRC or LEC transcripts and overlap between human and mouse. Identical TF transcripts between human and mouse are boxed.

Having identified medullary SC enrichment as a common feature of human and murine SI LNs, we asked how closely related these phenomena were at the cellular and predicted functional level. Human *SFRP2*+/*SFRP4*+, advSCs, *C7+* SCs, msLECs, and collect-LECs and mouse counterparts *Inmt*+, *Cd34+Cd248+*, or *Nr4a1*+ SCs, *Marco*+, and *Ptx3*+ LECs (Figure 2I; Table S2A) were the main cells to which SI LN-enriched SC transcripts were attributed. We examined which cell types expressed the most enriched transcripts in SI LNs (Figures 6A and 6B). In mice, ∼two-thirds were LEC-expressed, mostly by *Marco*+ cells, with the rest distributed across *Inmt*+/*Nr4a1*+, *Cd34+Cd248+*, and PvCs. In humans, half were LEC-expressed, each LEC subtype accounting for distinct transcripts. FRC-enriched transcripts were mainly shared between *SFRP2*+/*SFRP4*+ SCs, advSCs, and *C7+* SCs, with the highest in advSCs; angiotensinogen (AGT+) SCs accounted for ∼one-fifth of the transcripts. SI LN-enriched transcripts were associated with similar processes in both species, including vascular development, retinol metabolism, Wnt signaling, tissue remodeling, and organ development (Figure 6C). Pathway analysis showed that multiple processes intersect with each cell type, sometimes via single genes (Figures 6A and 6B), suggesting the entire medulla participates in these programs. The overlap of human and mouse DEGs (logFC > 0.5) between SI and LI LNs revealed 132 shared genes: ∼one-eighth of human and ∼one-third of mouse DEGs (Figure 6D), but >one-third human and half mouse GO pathways overlapped (Figure 6E). Further, analyzing which transcription factors (TFs) may be responsible for the DEGs enriched in SI LNs showed that the TFs formed networks that exhibited strong resemblance between mouse (Figure 6F) and human (Figure 6G), with most of the top 25 TFs being identical. scRNA-seq data allowed assignment of TFs to LECs or FRCs (Figures S6A and S6B), and most of the top 50 TFs were themselves DEGs (Tables S1C and S3C), confirming they are part of active networks in the LNs.

Therefore, these data suggest that human and mouse SI LN identity is governed by the enrichment of functionally similar cell types in the LN medulla and programmed by conserved TF networks.

### Differences between murine LNs along the gut manifest at weaning age, irrespective of the microbiota, but in dependence on dietary vitamin A

**Figure 7.**
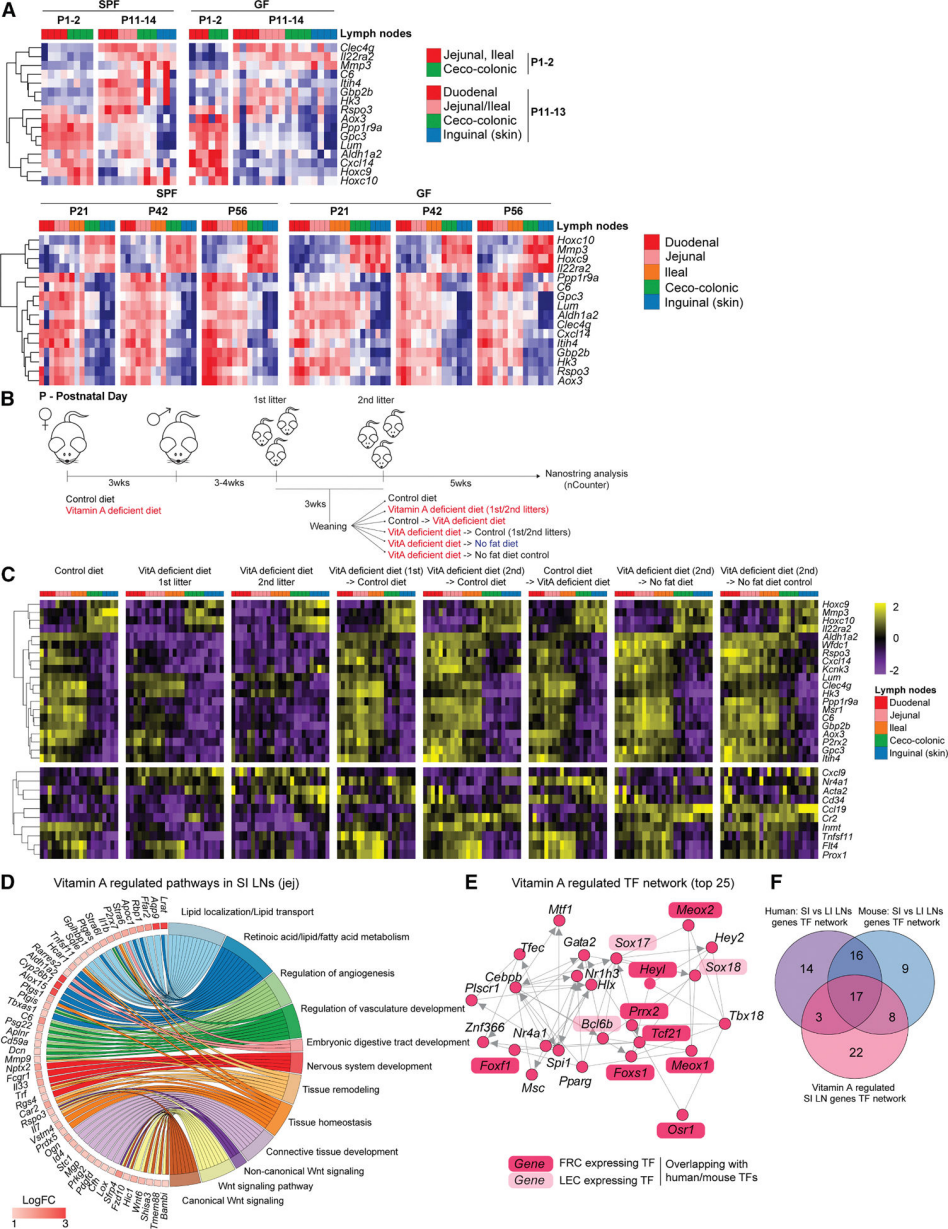
Differences between murine LNs along the gut manifest at weaning age, irrespective of the microbiota but in dependence on dietary vitamin A (A) Heatmap and hierarchical clustering of expression of indicated transcripts in indicated LNs from SPF or GF mice at P day 1–56 as measured by qPCR. *n* = 3–4, specified by the boxes on top of the heatmaps. (B) Schematic of the experimental setup to generate strictly vitamin A-deficient mice or not, and assessing the impact of changing diet at weaning (a total of eight experimental groups). (C) Heatmap and hierarchical clustering of expression of indicated transcripts in indicated LNs from the eight experimental groups from (B), as measured by NanoString. The separated bottom heatmap represents transcripts indicative of cell type representation: *Cxcl9* and *Ccl19*: T cell zone reticular cells (RCs); *Nr4a1*, *Inmt*, and *Cd34*: medullary sinus/cord RC; *Acta2*: perivascular RCs; *Cr2*: FDCs; *Tnfsf11*: subcapsular sinus RCs; *Flt4* and *Prox1*: LECs. *n* = 4–5. (D) Chord diagram connecting major GOBPs and DEGs (adjusted *p* value < 0.05) contributing to them that are enriched in the jejunal LNs of mice fed a vitamin A-sufficient control diet versus a vitamin A-deficient diet, based on bulk RNA-seq of jejunal LNs (*n* = 5). (E) Predicted TF network underlying DEGs between jejunal LNs from mice fed a vitamin A-sufficient control diet versus a vitamin A-deficient diet. TF genes are highlighted according to whether they regulate FRC or LEC transcripts and overlap with TFs regulating SI versus LI LN DEGs (Figure 6F). (F) Venn diagram showing the overlap between the number of TF predicted to regulate DEGs between human or mouse SI versus LI LNs and DEGs between SI (jejunal) LNs of vitamin A-sufficient versus -deficient mice.

We asked what drives the distinct medullary niche of SI LNs. Strong embryonic signatures suggested a developmental program, while lipid-related pathways indicated external influences, as SI LNs uniquely encounter lymph rich in chylomicrons, lipid-soluble vitamins, and bacterial products. In mice, natural environmental shifts coincide with weaning (days 14–21), when solid food intake rises, microbial biomass expands, and gut closure completes. We therefore analyzed the SI and LI LNs from newborn, 11-, 14-, 21-, 42-, and 56-day-old SPF and GF mice for our surrogate gene panel (Figure 3E). Newborn SI and LI LNs were indistinguishable (Figure 7A), but postnatal development differentially modulated transcripts, with some adult SI/ LI-enriched transcripts initially low in newborns. By postnatal (P)11–14, trends began resembling adult patterns, though not for all transcripts; inguinal LNs were already distinct from gut LNs. By P21, LNs matched adult patterns in both SPF and GF mice (Figure 7A), consistent with reports that postnatal LNs change cellular composition, with *Inmt+* and *Nr4a1*+ cells expanding by P28.^23^ Having confirmed the microbiota was not the main driver, we next asked whether dietary exposure influences the SI versus LI LN dichotomy. While suckling provides lipid-rich milk, access to lipid-soluble vitamins can be experimentally controlled via the mother’s diet. We observed an RA pathway enrichment (Figures 1D and 2C), and vitamin A exposure is known to support the tolerogenic potential of mLN SCs, which can also generate RA.^27^ We therefore asked whether vitamin A, to which SI LNs are more exposed than any other LNs through exposure to chylomicron-rich lymph,^2^ was involved. To test its role, dams were fed a vitamin A-deficient or -sufficient but otherwise isonutritional diet 3 weeks before breeding, and LNs of their first and second litters were compared at 8 weeks by NanoString (Figure 7B; Tables S6A and S6B). The first, and even more so the second litter, lost the SI LN-enriched gene expression, except for *Lum*, *Clec4g*, and *Hk3* (Figure 7C), while LI LN transcripts remained unchanged. To estimate how this correlated with changes in SC composition, we also designed a panel of SC subpopulation-specific marker transcripts and measured their expression (Figure 7C, bottom). SC marker analysis revealed the loss of *Inmt* and reductions in *Flt4*, *Prox1*, and *Tnfsf11*, indicating fewer *Inmt*+ SCs, LECs, and MRCs. Staining confirmed strongly reduced INMT+ areas in SI LNs, while LYVE+ areas were unaffected (Figure S7B). Diet swaps at 3 weeks or lowering vitamin A exposure postnatally restored SI LN gene expression and SC markers within 5 weeks (Figures 7B and 7C), demonstrating plasticity. In all these scenarios, vitamin A exposure overcame vitamin A deficiency, both as measured by the gain of the SI LN-enriched transcripts and the transcripts marking the SC populations. Since the gene panel analyzed under vitamin A deficiency was limited, we asked what fraction of all SI versus LI LN DEGs in SPF mice depended on vitamin A. To this end, we performed bulk RNA-seq of LNs from vitamin A-deficient versus sufficient mice (Tables S6C and S6D). Of the 108 top DEGs between jejunal and colonic LNs in SPF mice, 76 (∼75%) overlapped with vitamin A-regulated DEGs; 15 more trended down, and the rest increased in colonic LNs of deficient mice (Figure S7B; Tables S6F–S6H). Gene ontology biological process (GOBP) analysis of these DEGs (Figure 7D; Table S6E) resembled pathways enriched in human and mouse SI LNs (Figures 1D and 3C), and the associated TF network (Figure 7E) overlapped with the SI LN governing TFs. Of the top 50 TFs potentially regulated by vitamin A, 17 matched the human/mouse SI versus LI LN TFs (Figure 7F). Thus, vitamin A exposure explains most of the SI versus LI LN DEGs.

Taken together, these data identify dietary vitamin A as a powerful driving force for the development of the SI LN medulla.

## DISCUSSION

In this study, we uncover that human LNs are distinct depending on the organ they drain. Further, we describe that, within the gastrointestinal system, the most prominent characteristic of the SI LNs is an expanded medullary SC niche, while that of the LI LNs is active GCs. Furthermore, through systematically using similar approaches in mice, we discovered that these features are highly conserved between humans and mice. That LNs draining different organs possess a distinct architecture based on their SC composition has not been well characterized before in either species, but raises intriguing and important questions ranging from how this may influence adaptive immune outcomes,^13,19^ pathogen dissemination,^28,29^ or metastatic cell spread through the lymphatic system.^30,31^ The parallels also validate the mouse model as one suitable for predicting driving forces and consequences of immunologically distinct LNs in the digestive system of humans. As such, we identified dietary vitamin A as a major orchestrator for murine SI LN identity that, based on transcriptional network conservation, is likely also a factor shaping human SI LN composition.

Our technical approach to perform bulk RNA-seq on undisturbed tissue revealed an aspect of LN identity, essentially different SC architecture, that would probably have escaped approaches relying on prior tissue digest. It also permitted the least biased comparison between human and murine LNs. We were initially surprised by the signatures that distinguished SI and LI LNs, as we had expected profiles linked more directly to immune cell functions and a tolerogenic versus more pro-inflammatory and active signature.^2^ Our strategy also highlights the merit of using published scRNA-seq datasets as a resource. Further, we established a framework for the quantification of cells based on tissue sections of asymmetric organs like an LN, whereby it is crucial to aim for mounting a sample along any symmetry axis it may have and stain multiple sections coming from throughout the tissue to avoid biases and false conclusions. Finally, in the advent of global spatial transcriptomics, including LNs, our whole LN RNA-seq datasets provide a crucial reference point to verify if cellular distributions found are true or biased by the cuts analyzed.

The LN medulla has several ascribed roles, ranging from DC maturation,^32^ plasma cell survival,^33^ and a source of structural and matrix-producing cells^34^ to lymphocyte imprinting during exit.^35^ The existence of multiple SC subsets in LNs has only recently been discovered, and while roles for SCs in the T and B cell zones have been more defined,^36–38^ the medullary SC subsets still lack clear functional assignments. The conservation of a medulla enriched in SCs in SI LNs suggests that this niche has a similar and important purpose in humans and mice. Of the medullary SC populations we found responsible for the SI LN signature, *Cd34*+*Cd248+* cells originate from *Cd34+*, while *Inmt*+ and *Nr4a1*+ cells from FRC precursors and *S100a4*+ cells from both.^23^ *Cd34*+ SCs have been ascribed a stem cell-like role that may serve *in situ* to replace and expand SCs,^13,23^ found to be responsible for maintaining LN capsule integrity^13^ and to support (lymph) angiogenesis and tissue repair.^39,40^ A subset of CD34^+^ cells enriched in mLNs versus skin LNs also expresses *Aldh1a2*^13^ and has been proposed to contribute to the tolerogenic environment of gut LNs.^41^ Of note, the reference dataset we used did not recover this subset. Based on the pathways and transcripts enriched in *Inmt*+ and *Nr4a1*+ cells, they seem to be involved in the generation of and composition of the extracellular matrix (e.g., *Dcn*, *Lum*, *Itih4*, and *Ppp1r9a* among our SI versus LI LN DEGs) or promote differentiation and proliferation (e.g., *Gpc3* and *Rspo3*). However, with the field so young, none of these roles have been confirmed by ablation of medullary SCs. Medullary LECs have been shown to act as a firewall for viruses,^42^ attract neutrophils,^7^ together with subcapsular LECs act as a reservoir for lymph-borne antigen,^43^ and, overall, guide lymphocytes out of the LN through the generation of a sphingosine 1 phosphate gradient.^44^ Among the LN macrophages, more is known about SSMs, which are thought to catch and present soluble antigens to B cells in underlying B cell follicles and act as first responders for lymph-borne pathogens.^24,45^ MSMs distinguish themselves by being highly phagocytic, based on their fluorescent antigen capture,^46^ and likely have at least a similar capacity to SSMs to target pathogens. *C6* and *Gbp2b*, which are confirmed to be enriched in MSMs, are involved in bacterial containment. More specific targeting of SSMs versus MSMs will inform whether they serve similar roles, just in different niches, or have distinct functions. Remarkably, medullary LECs and macrophages share some hallmark genes, such as *Marco* and *Lyve1*, suggesting they are either imprinted as a niche or act in concert using similar pathways.

The co-enrichment of FRCs, LECs, and MSM in the SI LN medulla raises two fundamental, intriguing questions: do these cell types communicate with each other to coordinate the niche’s function, and if so, which signaling axes are most important for this? What is the purpose of the seemingly unique expansion of these cells in the SI LNs? As to the latter, we can only speculate, but unique challenges to the SI LNs are high lymph flow rates (the majority of consumed water is absorbed in the small intestine,^47^ the postprandial flow rate is elevated,^48^ and intestinal lymph accounts for about 80% of thoracic duct lymph^49^), exposure to highly concentrated chylomicrons and other lipid-soluble molecules, and exposure to dietary antigens. They are also the first LNs to see orally ingested pathogens that make it beyond the mucosa. An extensive medullary network may thus allow for easing fluid pressure, stem cell-like cells allow for faster replenishment of cells damaged by chyle exposure, and dietary antigen retention ensures efficient tolerance induction.

That all these functions may be in part orchestrated by vitamin A, itself a molecule carried by lymph and often chylomicrons, is compatible with such a proposal. The dependence on a vitamin may seem risky, but the vitamin A metabolite retinoic acid (RA) is a generic morphogen,^50^ and within the mucosal immune system has pleiotropic functions such as induction of peripheral regulatory T cells and IgA+ B cells.^51–54^ In the future, it will be interesting to decipher to what extent changes in the immune compartment are mediated through direct RA signaling in hematopoietic cells or indirectly via RA’s effects on SCs, using cell-type-specific interference with the RA receptors. Our finding that the microbiota largely played no role in setting up the difference between the SI and LI LNs is in line with reports showing that *Cd34+* cells are enriched in mLNs, and they and *Inmt*+ and *Nr4a1*+ cells are independent of the microbiota in their relative abundance.^13^ The tuning of the amplitude of the DEGs between SI and LI LN in GF mice seen in our RNA-seq dataset could indirectly be caused by the SI microbiota’s role in lipid absorption,^55^ which by extension could also affect vitamin A absorption.

In sum, our data identify the LN medulla as a niche that warrants regionally aware investigation. Since LNs expand and contract during infection and disease, understanding how medullary cell types adapt could open opportunities for targeted manipulation.

### Limitations of the study

Our analysis focused on features unique to SI LNs and their drivers, leaving open questions about differences between lung and gut LNs or drivers of LI LN profiles. Whole LN bulk RNA-seq captures the largest differences, favoring transcripts reflecting cell number, while subtle per-cell expression differences or differences originating from less abundant cells are harder to detect.^2^ Gene-to-cell type assignments were limited by the coverage and resolution of existing scRNA-seq datasets. Notably, the strong neuronal gene signature in SI LNs suggests differences in LN innervation, but scRNA-seq of LN neurons is only available for mice.^56^

## RESOURCE AVAILABILITY

### Lead contact

Further information and requests for resources and reagents should be directed to and will be fulfilled by the lead contact, Daria Esterhá zy (desterhazy@bsd.uchicago.edu).

### Materials availability

No new material was generated in this study.

### Data and code availability

- Bulk RNA-seq data are available in the GEO database under the following accession numbers: Human lung/gut lymph nodes: GEO: GSE281776; SPF/GF mouse lymph nodes: GEO: GSE281824; *Rag1*^−/−^ mouse intestinal lymph nodes: GEO: GSE282128; vitamin A-deficient diet-fed B6 mouse intestinal lymph nodes: GEO: GSE281117; LN CD169 macrophages: GEO: GSE281209; and LN-sorted macrophages: GEO: GSE282129.
- This paper does not report original code.
- Any additional information required to reanalyze the data reported in this work paper is available from the lead contact upon request.

## STAR★METHODS

### EXPERIMENTAL MODEL AND STUDY PARTICIPANT DETAILS

#### Animals

All mouse studies were approved by The University of Chicago Institutional Animal Care and Use Committee. Mice were maintained at the University of Chicago animal facilities under SFB-free specific pathogen-free (SPF) conditions. B6 (C57BL/6J), *Rag1*^−/−^ (B6.129S7-Rag1^tm1Mom^/J), RiboTag (B6J.129(Cg)-Rpl22^tm1.1Psam^/SjJ) mice were purchased from the Jackson Laboratories and maintained in-house. Germ-free (GF) mice were purchased from Taconic and maintained in isolators in the gnotobiotic facility at the University of Chicago. In this study, ex-GF (SPF) mice are the offspring of GF mice taken out of isolators and colonized with a microbiome from JAX mice with the same bedding and food. CD169-Cre, B6-Siglec1/Cre KI mice were obtained from Dr. Masato Tanaka, Tokyo University of Pharmacy and Life Sciences, School of Life Science. Male and female mice were used for this study, and the animals were 0–8 weeks of age. All mice were on the C57BL/6J background and were bred in-house.

#### Human specimens

Human gut and lung-draining lymph node samples were obtained through the Gift of Hope organ donor program (Drs. Jonathan Kent and Lucia Madariaga). The study was approved under IRB 22–0501.

### METHOD DETAILS

#### Human lymph node isolation

To study the regionalization of human gut-, and lung-draining lymph nodes, the following lymph nodes were isolated (Figure 1A). Intestine-draining: superior central, mesenteric, juxta-intestinal, ileocolic, caecum, ascending colon; lung-draining: right lower paratracheal (4R), subcarinal (7), right hilar (10R), right subsegmental (14R).

#### Mouse lymph node isolation

Gut (duodenum, jejunum, ileum, caecum-colon, iliac), celiac, liver, mediastinal (lung), inguinal, popliteal, axillary, deep cervical, superficial cervical lymph nodes were excised, places in ice-cold PBS, and any remaining afferent or efferent lymphatic vessels and epinodal fat were removed prior to subsequent analyses.

#### Dietary interventions

To study the role of Vitamin A on gut-draining lymph nodes regionalization, 7–8 weeks old C57BL/6J females were placed on a Vitamin A deficient (TD.86143, Inotiv) or ingredient matched control with Vitamin A added back at 20 IU/g (TD.91280, Inotiv) 3 weeks before breeding set up. First and second litters were sacrificed at age 3 or 8 weeks old. 8-week-old mice were placed on different diets at the weaning stage (week of 3): pups from the female and male fed with vitamin A deficient or sufficient diet were put on vitamin A sufficient or deficient diet respectively or stayed on the same diet they were on before weaning or fed with no fat diet (TD.180890, Inotiv, 0.5% of calories from fat, which come from the grain shells), or no fat diet control (CHOW, Inotiv, 16% of calories from fat) (Figure 7B scheme).

#### Flow cytometry analysis of lymph node macrophages

Lymph nodes were isolated and placed into 200 μL cold RPMI, supplemented with 5% FCS and 1% HEPES (dissection medium). Tissues were chopped and digested using 2.5 mg/mL Collagenase D for 30 min at 37^◦^C. The reaction was stopped by placing the digest on ice. Cells were spun down at 700g for 5 min at 4^◦^C, topped up with medium again, and then subjected to staining. Cells were first stained for viability using Near Red Dead stain (1:500, Invitrogen) followed by surface markers staining In FACS buffer (PBS, 1% BSA, 2 mM EDTA, 0.02% NaN_3_) for CD11b (BV421), CD8a (BV605), TCRb (BV711), B220 (BV711), F4/80 (FITC), MHCII (PercpCy5.5), CD11c (AF647), NK1.1 (Cy7APC), CD90 (Cy7APC), CD103 (PE), and CD169 (Cy7PE) (all at 1:200, except for MHCII, which was at 1:8000). Flow cytometry was conducted on an LSRII (BD Biosciences) and analyzed using FlowJo Software.

#### Lymph node macrophages sorting

To sort macrophages from SPF B6 (C57BL/6J) mice, cells were pregated as live, CD45+TCRb–B220–NK1.1–IL7R–CD90–CD11c^int^MHCII^int^CD11b+ and then gated as CD169+F4/80–(subscapular sinus (SSM)), CD169+F4/80+ (medullary sinus (MSM)), and CD169–F4/80+ (medullary cord (MCM)) macrophages from duodenum, jejunum, ileum, cecum-colonic, inguinal LNs at the University of Chicago Flow Cytometry Core using a BD FACSAria Fusion Cell Sorter. Cells were sorted into TCL buffer containing 1% of 2-mercaptoethanol, spun down, and frozen on dry ice. Samples were stored at −80^◦^C until the processing.

#### Flow cytometry analysis of human/mouse lymph nodes GC B cells

Human lymph nodes were isolated and placed in 1000 μL cold RPMI supplemented with 5% FCS and 1% HEPES (dissection medium). Tissues were chopped and digested using 2.5 mg/mL Collagenase D for 40 min at 37^◦^C. Cells were filtered using the 70 μl cell strainer into 15mL tubes, spun down at 700g for 5 min at 4^◦^C, topped up with medium again, and then subjected to staining. Cells were first stained for viability using Live/dead Fixable Blue Dead cell stain kit (1:500, Invitrogen) followed by surface markers staining in FACS buffer (PBS, 1% BSA, 2 mM EDTA, 0.02% NaN_3_) for CD95 (BB515), CD3 (BV480), CD45RA (BV570), and CD19 (eFluor506) (all at 1:100).

Mouse lymph nodes were isolated and placed into 200 μL cold RPMI (described above). Tissues were homogenized by mechanical disruption between two frosted microscopy slides to obtain a single-cell suspension and filtered through a 70 μm mesh into the 96-well plate. Cells were spun down at 700 g for 5 min at 4^◦^C, topped up with medium again, and then subjected to staining. Cells were first stained for viability followed by surface markers staining in FACS buffer (PBS, 1% BSA, 2 mM EDTA, 0.02% NaN_3_) for CD95 (BV421), B220/CD45RA (BV711), CD3^−^, CD4^−^, CD8^−^, NK1.1 (BV785/BV786 to exclude non-B cells populations (all at 1:200). Flow cytometry was conducted on a Cytek Aurora (Cytek Biosciences) for both human and mouse and analyzed using FlowJo Software.

#### Mouse/human tissue (lymph node) immunofluorescent staining

Mouse intestine (duodenum, jejunum, ileum, caecum-colon) draining lymph nodes and skin draining inguinal lymph nodes were excised, frozen in an Optimal Cutting Temperature (O.C.T.) compound (Tissue Tek (Sakura)) on dry ice and stored at −80^◦^C. Ten-micrometer frozen tissue sections were cut in intervals of 50–100 μm using a cryostat.

Human lymph nodes were fixed in 4% freshly prepared PFA in 1× PBS overnight in a cold room and transferred to 70% ethanol. They were then submitted to the University of Chicago Human Tissue Resource Center for paraffin embedding and cutting of 10 μm sections.

Mouse frozen 10 μm lymph node sections were fixed for 15 min in 4% PFA at RT and incubated for 1h in 0.1% Triton X-100 (Thermo Fisher) in PBS (PBS-T) followed by incubation with blocking buffer (1% BSA, 5% donkey serum in PBS-T).

Human paraffin-embedded sections were melted at 70^◦^C and rehydrated in xylene for 10 min x 2 and the following ethanol series: 100%, 95%, 85%, 70%, and 50%–2 times for 3 min each. Slides were washed in ddH2O, followed by 1× PBS, and incubated in permeabilization/blocking buffers for 30 min each.

The following primary antibodies were used for immunofluorescence staining of mouse tissue: goat polyclonal anti-Lyve-1 (1:200), rat monoclonal anti-CD45R (B220) (1:100), rabbit anti-CD3 (1:100), rabbit monoclonal anti-TEMT (INMT) (1:100), anti-HA(1:200), antiF4/80 (1:100), anti-CD169 (1:100) and for human tissue: goat polyclonal anti-human LYVE1 (1:100), rabbit monoclonal anti-human DCN (1:100). Alexa Fluor secondary antibodies donkey anti-goat 488, donkey anti-goat 568, donkey anti-rabbit 488, and donkey anti-goat 568 (1:500) were used for visualization. Whole slide images were taken with an Olympus VS200 Research Slide Scanner (Olympus/Evident, Center Valley, PA) with a Hamamatsu ORca-Fusion camera (Hamamatsu Photonics, Skokie, IL). Individual images were visualized using the QuPath software v0.5.1.

#### RNAscope analysis in combination with antibody staining

RNAscope analysis of fresh-frozen 10 μm lymph node sections was performed using the RNAscope Multiplex Fluorescent Detection Reagents v2 kit according to the manufacturer’s instructions (Advanced Cell Diagnostics). The RNAscope probes used targeted *Inmt* (486371-C2), *Lum* (480361-C3), *Clec4g* (503371-C2), and *Hk3* (1055121-C2). After mRNA staining, slides were washed in 1× PBS and incubated in permeabilization buffer (0.1% Triton in PBS), followed by blocking buffer (1% BSA, 5% normal donkey serum in permeabilization buffer). The primary antibodies against Lyve-1 and CD3 cells were used to co-stain with mRNA.

#### Histological analysis of human/mouse whole LN sections

Digital whole-slide z stack.vsi image files were imported and analyzed using QuPath v0.5.1. For LEC and FRC quantification in human and mouse lymph nodes, regions of interest were manually outlined using the freehand selection tool. LYVE1^+^ DAPI^+^ cells (LECs in both human and mouse), DCN^+^ DAPI^+^ cells (human FRCs), and INMT^+^ DAPI^+^ cells (mouse FRCs) were identified and quantified using the ‘Positive Cell Detection’ function. Cell density (cells/mm^2^) was calculated and used for comparison. Quantification was based on an average (mean) of 4–6 sections per human lymph node (spaced 500–600 μm apart) and 3–4 sections per mouse lymph node (spaced 100–200 μm apart).

For each human lymph node, 4–6 sections were analyzed at ∼500–600 μm intervals. Samples were obtained from four human donors. For DCN analysis, one small intestine (SI) and one large intestine (LI) lymph node were collected from each donor (total = 4 LNs per group). For LYVE1 analysis, two SI and two LI lymph nodes were collected from one donor, and one SI and one LI lymph node from the remaining donors (total = 5 LNs per group). Donor information is provided in Figure S1A.

#### RNA extraction (exGF, GF, and *Rag1*^−/−^ mice LNs) for sequencing

Lymph nodes were isolated, snap-frozen on dry ice in 1.5 mL Eppendorf tubes with 0.3–0.5 mL of RNAlater (Sigma) and stored at −80^◦^C until processing. Samples were defrosted and transferred into 2 mL Bead Mill Tubes pre-filled with ceramic beads and 1 mL cold TRIzol Reagent (Thermo Fisher) followed by lysis using Omni Bead Ruptor (speed – 2.90–3.10, cycles – 2, time per cycle – 30 s). Samples were transferred to a new tube and processed according to the manufacturer’s instructions. Briefly, the samples were incubated at RT and then spun down at 4^◦^C, and the supernatant was transferred to a new tube with chloroform to extract the RNA and spin down again. The RNA was precipitated by adding equal volumes of 70% EtOH and continuing the RNA purification process with the PicoPure RNA Isolation Kit (Thermo Fisher) according to the manufacturer’s instructions and eluting in 20–30 μL of the elution buffer. RNA samples were purified using RNAclean XP beads (Beckman Coulter), and RNA concentration was measured with a NanoDrop Microvolume Spectrophotometer (Thermo Fisher).

#### RNA extraction (human, vitamin A-deficient diet-fed mice LNs) for sequencing

Lymph nodes were isolated and placed into 1.5 mL Eppendorf tubes with 0.3–0.5 mL of *RNAlater* and stored at − 80^◦^C until processing. Samples were defrosted and homogenized with RNAse/DNase-free 2.8 mm Ceramic Beads using Omni Bead Ruptor (speed – 2.90 (mouse), 4.00 (human), cycles – 2, time per cycle – 30 s) in 1 mL of TRIzol. Homogenized samples were transferred into the new Eppendorf tubes, and 200 μL of Chloroform was added, tubes were shaken by hand and centrifuged at 12,000 g for 15 min, 4^◦^C. The aqueous phase was transferred to the new tubes, and an equal volume of isopropanol was added and resuspended. Samples were incubated overnight at 80^◦^C. On the second day, samples were centrifuged at 10,000 g for 20 min, 4^◦^C, washed in 500 μL of 75%-fresh-prepared ethanol, and centrifuged at 10,000 g for 5 min, 4^◦^C. Pellets were air-dried for ∼5 min and resuspended in 20 μL (mouse LNs) and 50 μL (human LNs) in Ultrapure DNase/RNase Free Distilled Water. RNA samples were purified using RNAclean XP beads (Beckman Coulter), and RNA concentration was measured with a NanoDrop Microvolume Spectrophotometer (Thermo Fisher).

#### RNA extraction and gene expression analysis by quantitative real-time PCR

Lymph nodes were isolated and placed into 1.5 mL Eppendorf tubes with 0.3–0.5 mL of *RNAlater* and stored at − 80^◦^C until processing. Samples were defrosted and homogenized with RNAse/DNase-free 2.8 mm Ceramic Beads using Omni Bead Ruptor (speed – 3.10, cycles – 2, time per cycle – 30 s). RNA was extracted using TRIzol manufacturer’s protocol. 500–1000 ng of RNA was used as an input and cDNA was prepared with the SuperScript IV Reverse Transcriptase system according to the manufacturer’s protocol. After completion, cDNA was diluted with RNase-free water (1:5 (for ∼500 ng input) or 1:10 (for 1000 ng input). Gene expression was analyzed by qPCR master mix using Power Sybr Green (Invitrogen) containing 10 ng cDNA, 7.5 μL Sybr Green, 1.5 μL 10 μM forward and reverse primer mix, and water up to 15 μL. Gene expression analysis was performed using the Applied Biosystems QuantStudio 6 Flex system. Duplicate samples were loaded into 384-well plates, and relative gene expression levels were normalized to *38b4* housekeeping gene expression using the 2^–ΔCT method, with gene expression values displayed representing the POWER (2^–ΔCT) × 10,000.

#### Lymph node CD169-macrophages mRNA isolation (immunoprecipitation)

RiboTag mice were crossed to a CD169-Cre recombinase-expressing mouse to activate the *Rpl22*^*HA*^ allele in all tissues. Homozygous CD169^RPL22HA^ mice lymph nodes were collected and used for the RiboTag immunoprecipitation according to the protocol (http://depts.washington.edu/mcklab/RiboTagIPprotocol2014.pdf) with the following modifications: samples were homogenized using Omni Bead Ruptor (speed – 3.50, cycles – 2, time per cycle – 30 s) in 0.5 mL supplemented homogenization buffer and processed with modifications described before by Paul A. Muller et al. (2020).^58^ RNA was extracted using the Arcturus PicoPure RNA isolation kit (Applied Biosystems) according to the manufacturer’s instructions.

#### *RNAseq* library preparation (exGF, GF, *Rag1*^−/−^, vitamin A deficient diet-fed mice, human LNs, CD169^RPL22HA^ macrophages)

1000–1500 μg of RNA was used as the starting material for cDNA library synthesis and tagmentation using the NEBNext Poly A kit (NEB) per the manufacturer’s instructions. The concentration of the eluted samples was measured using a Qubit Fluorometer (Thermo Fisher) and the average fragment length of 300–400 bp was confirmed with a Bioanalyzer (Agilent). Samples were pooled at 10 nM and sequenced using paired-end 50 bp reads on a Novaseq S2 flowcell at the University of Chicago Functional Genomics Core. Vitamin A deficient diet fed mice lymph nodes, and CD169^RPL22HA^ macrophages RNA libraries were sequenced on a NextSeq2000 at the University of Chicago Single Cell Immunophenotyping Core.

#### *RNAseq* library preparation (sorted LN macrophages)

Following RNA extraction and purification with Clean XP beads (Beckman Coulter) reverse transcription was performed using following primers: P1-RNA-TSO: Biot-rArArUrGrArUrArCrGrGrCrGrArCrCrArCrCrGrArUrNrNrNrNrNrNrGrGrG, P1-T31: BiotAATGATACGGCGACCACCGATCG31T, P1-PCR: Biot-GAATGATACGGCGACCACCGAT. RNA was eluted for 1 min in RT-cDNA synthesis mix containing (0.5 μL P1-T31 (20 μM), 0.3 mL RNasinplus (Promega), 1.5 μL 10 μM dNTP, 3.5 μL 10 μM Tris pH 7.5–0.5% IGEPAL CA-630 (Sigma) and 1.7 μL RNase free ddH_2_O). The eluted RNA was incubated for 3 min at 72^◦^C, then combined with 7.5 μL of reverse transcription mix (3 μL 5× RT Buffer, 0.375 μL 100 mM DTT, 0.375 μL RNasin plus, 0.5 μL P1-RNA-TSO (40 μM), 0.75 μL Maxima RT Minus H (Thermo Scientific), 1.8 μL 5M Betaine (Sigma), 0.9 μL 50 mM MgCl_2_ and 0.175 μL RNase free ddH_2_O). Samples were subjected to the following PCR reaction protocol: 42^◦^C for 90 s, (50^◦^C for 2 min, 42^◦^C for 2 min) ×10 cycles, 70^◦^C for 15 min. The cDNA was then amplified using 13.5 μL cDNA, 20 μL KAPA HiFi 2× Mix, 1.5 μL P1-PCR, and 5 μL H_2_O. Samples were subjected to the following protocol: 98^◦^C for 3 min, (98^◦^C for 15 s, 67^◦^C for 20 s, 72^◦^C for 6 min) for ×12 cycles, and 72^◦^C for 5 min cDNA was cleaned using RNA Clean XP beads and eluted in nuclease-free water. Qubit fluorometer was used to measure the concentration and the average fragment length of 1500–1800 was determined by Bioanalyzer. Samples were normalized using ddH_2_O, and 2.5 μL cDNA was tagmented using Nextera XT Index Kit according to the manufacturer’s protocol, except that all volumes were used at 0.5× of the indicated volumes. Samples were pooled and sequenced using paired-end 50 bp reads on a Novaseq S2 flowcell at the University of Chicago Functional Genomics Core.

#### RNA sequencing analysis

Raw FastQ files were pseudo-aligned to the mouse or human reference transcriptome (GRCm39 or GRCh38.p13, respectively) using Kallisto.^59^ The gene counts were input to R and transcripts per million (tpm) were scaled by gene length. Initial filtering removed transcripts with expression under 1 count per million (cpm). Filtered gene counts were used as an input for differential expression analysis using limma/voom (edgeR). Genes with an adjusted *p*-value (adj_pval) < 0.05 were considered significant for downstream analysis. ImmGen analysis was performed using https://rstats.immgen.org/MyGeneSet_New/index.html databrowser and the top 200 upregulated or down-regulated transcripts between SI and LI LNs were taken for the analysis of expression across ImmGen cell types. Principal component analysis (PCA) was performed using normalized counts per million (cpm) values using pca3d v0.10.2 and rgl v1.3.12 packages. Heatmaps were generated using the total DEG list (log_2_ fold changes (logFC) greater than 1.5 or less than − 1.5 for human LNs, 1.5 < logFC < − 1.5 for SPF or GF mice LNs, 1.45 < logFC < − 1.45 for *Rag1*^− /−^ mice LNs, 1.2 < logFC < − 1.2 for Vitamin A deficient/sufficient fed mice and CD169^RPL22HA^) using the R package pheatmap v1.0.12.

Gene ontology (GO) analysis was performed using clusterProfiler v4.12.0 (DEG list: log_2_ fold changes greater than 0.5 for up-regulated or less than − 0.4 for down-regulated pathways for human superior central versus caecal and SPF mouse jejunum versus colon LNs, all DEGs with adjusted *p* value less than 0.05 for SPF versus GF mice jejunum LN and Vitamin A sufficient versus deficient dietfed mice jejunum LN). GOplot v1.0.2 GOchord function was used to visualize selected GO biological process (BP) and molecular function (MF) pathways. The transcription factor (TF) network and associated analysis were performed by putting all DEGs with an adjusted *p*-value <0.05 into the ChEA3 search database. The top 50 TFs were taken for downstream analysis (comparison between humans, exGF mice, and mice fed with Vitamin A deficient diet). Venn diagrams comparing differentially expressed gene (DEG) lists and TFs between datasets were generated using the R package Venn Diagram v1.7.3. For human and mouse DEG comparison, mouse transcripts were converted to their human equivalent using getLDS() function in biomaRt v2.60.1 package. To show the percentage of cells expressing DEG (pie charts, Figures S2C and S2D), up-and down-regulated transcripts with the logFC greater than 1.4 and lower than − 0.4, respectively, were used for the DotPlot visualization with the following extraction of input file with the average expression and cell type ID info. Genes with an average expression scaled greater or equal to 0.5 and an average expression ≥0.1 were used to count the number of transcripts corresponding to a particular cell type ID.

For CD169^RPL22HA^ analysis, initial filtering removed transcripts with expression higher than 3 count per million (cpm) in isotype control (to filter out non-CD169 transcripts), and the rest of the transcripts were filtered by removing transcripts under 3 cpm. To show the macrophage-expressing transcripts across three different LN macrophage cell types (SSM, MSM, MCM), the transcripts after CD169^RPL22HA^ filtering were taken for boxplot generation (Figure 5G).

#### Analysis of single-cell datasets

A published single-cell RNA sequencing data was re-analyzed, UMAP plots were generated according to the author’s provided method description, and the expression analysis of DEG in human and mouse lymph node cell populations was performed using publicly available *scRNAseq* from Y. Abe et al. (2022),^6^ A. Takeda et al. (2019),^7^ De Martin and Stanossek et al. (2023),^20^ Lenti et al. (2022),^23^ and Tong and Mang et al. (2023).^18^ Specific gene expression was plotted using FeaturePlot, using a blend to overlay two transcripts. Gene set (DEG) expression analysis was performed using addModuleScore function followed by FeaturePlot. To show cellular and functional similarities between human and mouse lymph nodes DEG (mouse: Inmt+, Cd34+Cd248+, Mgp+ PvC, Nr4a1+, Marco+, Ptx3+ cells; human: advSC, AGT, SFRP2/SFRP4+, C7+, bLEC, cLEC, collectLEC, fLEC & pfsLEC, msLEC), with the average expression scaled (avg.exp.scaled) ≥ 0.5 and average expression ≥0.1 per *scRNAseq* data was taken out for the DotPlot visualization (Figures 6A and 6B) and associated common GOBP pathways were indicated.

#### NanoString (nCounter) gene expression analysis

mRNA from Vitamin A deficient/sufficient diet-fed mice LNs was analyzed using NanoString nCounter Gene Expression Assay to detect the expression of 35 transcripts in a single reaction. Expression analysis was conducted at the Human Immunologic Monitoring (HIM) facility at the University of Chicago. 100ng RNA input was used per sample, and the analysis was performed according to the manufacturer’s recommended protocol (NanoString Technologies; Seattle, WA). mRNA was quantified using the nCounter Digital Analyzer as counts and data were normalized to the following housekeeping transcripts *Actb, Polr1b, Rpl19, Rplp0, Tbp* using the nSolver software (NanoString Technologies, Seattle, WA).

### QUANTIFICATION AND STATISTICAL ANALYSIS

RNA sequencing data were analyzed using R software, including statistical analysis. For human DEG normalization to LEC and FRC marker transcripts, human DEG counts per million (CPM) between small and large intestine draining LNs were normalized to *PROX1* and *DCN,* respectively, and adjusted or non-adjusted *p* values were calculated using stats (v. 3.6.2) t test function. Other data were analyzed using Prism software (GraphPad). Data are presented as the mean ± SEM. A two-way ANOVA, paired t test, or unpaired t test was used for comparisons. Numeric *p*-values or “ns” are noted when the value is greater than 0.05 and considered non-significant; asterisks indicate *p*-values are noted when differences are less than or equal to 0.05 and are considered significant. *p < 0.05, **p < 0.01, *** *p* < 0.001, ****p < 0.0001).

## Supplementary Material

1

2

3

4

5

6

7

Supplemental information can be found online at https://doi.org/10.1016/j.celrep.2025.116441.

## Figures and Tables

**Table:** KEY RESOURCES TABLE

REAGENT or RESOURCE	SOURCE	IDENTIFIER

Antibodies		
Rabbit monoclonal anti-mouse TEMT (INMT)	Abcam	ab181854
Goat polyclonal anti-mouse LYVE1	R&D systems	AF2125; RRID: AB_2297188
Rat monoclonal anti-mouse B220/CD45R	R&D systems	MAB1217; RRID: AB_357537
Goat polyclonal anti-human LYVE1	R&D systems	AF2089; RRID: AB_355144
Rabbit monoclonal anti-human DCN (Alexa Fluor 647)	Abcam	ab281326–100UL; RRID: AB_3678775
Rat monoclonal anti-mouse/human CD45R (B220)	eBioscience	14–0452-82; RRID: AB_467254
Rabbit polyclonal anti-mouse CD3	Abcam	ab5690; RRID: AB_305055
Rat monoclonal anti-mouse Cd169	Bio-Rad	MCA884; RRID: AB_322416
Rat monoclonal anti-human/mouse CD11b (Brilliant Violet 421)	BioLegend	101251; RRID: AB_2562904
Rat monoclonal anti-mouse CD8a (Brilliant Violet 605)	BioLegend	100744; RRID: AB_2562609
Armenian Hamster anti-mouse TCRb chain (Brilliant Violet 711)	BioLegend	109243; RRID: AB_2629564
Rat monoclonal anti-mouse CD45R/B220 (Brilliant Violet 711)	BioLegend	103255; RRID: AB_2563491
Rat monoclonal anti-mouse F4/80 (Alexa Fluor 488)	BioLegend	123120; RRID: AB_893479
Rat monoclonal anti-mouse/human CD11c (Alexa Fluor 647)	BioLegend	101218; RRID: AB_389327
Mouse monoclonal anti-mouse NK1.1 (APC/Cyanine7)	BioLegend	108724; RRID: AB_830871
Rat monoclonal anti-mouse CD169 (Siglec-1) (Alexa Fluor 594)	BioLegend	142416; RRID: AB_2565620
Rat monoclonal anti-mouse MHC Class II (I-A/I-E) (PerCP-eFluor 710)	Invitrogen	46–5321-82; RRID: AB_1834439
Rat monoclonal anti-mouse CD90.2 (APC-Cy7)	BD Biosciences	561641; RRID: AB_10898013
Rat monoclonal anti-mouse CD103 (PE)	BD Biosciences	557495; RRID: AB_396732
Rat anti-mouse CD8 (BV786)	BioLegend	100749; RRID: AB_11218801
Rat anti-mouse/human CD45RA/B220 (BV711)	BioLegend	103255; RRID: AB_2563491
Hamster anti-mouse CD95 (BV421)	BD Biosciences	562633; RRID: AB_2737690
Rat anti-mouse CD3 (BV785)	BioLegend	100231; RRID: AB_11218805
Rat anti-mouse CD4 (BV786)	BD Biosciences	563727; RRID: AB_2728707
Rat anti-mouse NK1.1 (BV785)	BioLegend	108749; RRID: AB_2564304
Mouse anti-human CD95 (BB515)	BD Biosciences	564597; RRID: AB_2744470
Mouse anti-human CD3 (BV480)	BD Biosciences	566166; RRID: AB_2739563
Mouse anti-human CD19 (eFluor 506)	eBioscience	69–0199-42; RRID: AB_2637384
Mouse anti-human CD45RA (BV570)	BioLegend	304131; RRID: AB_10897946
Donkey Anti-Rat IgG Antibody (Alexa Fluor^®^ 647)	Jackson ImmunoResearch	712–605-153; RRID: AB_2340694
Donkey Anti-Goat IgG Antibody (Alexa Fluor^®^ 488)	Jackson ImmunoResearch	705–546-147; RRID: AB_2340430

Biological samples		

Human intestine/lung lymph nodes	Gift of Hope organ donor program	IRB 22–0501

Chemicals, peptides, and recombinant proteins		

Trizol	Life Technologies	15596018
Chloroform	Fisher Scientific	BP11451
2 Propanol (Certified ACS), Fisher Chemical	Fisher Scientific	A416 4
Heparin sodium salt from porcine intestinal mucosa	Sigma-Aldrich	H3393–50KU
Rnalater^®^, stabilize and protect RNA with immediate RNase inactivation	Sigma-Aldrich	R0901–500ML
RNasin Plus RNase Inhibitor	Promega	N2615
Dithiothreitol (DTT, White Crystals or Powder/Electrophoresis)	Fisher Scientific	BP17225
Invitrogen ambion KCl (2M)	Thermo Fisher Scientific	AM9640G
DNase I	Millipore Sigma	10104159001
Magnesium chloride solution, for molecular biology	Sigma-Aldrich	M1028–100ML
10% NP-40	Abcam	ab142227
Cycloheximide, ≥93.0% (HPLC)	Sigma-Aldrich	01810–1G
EDTA-free Protease Inhibitor Cocktail	Millipore Sigma	11873580001
UltraPure 1 M Tris HCI Buffer, pH 7.5	Thermo Fisher Scientific	15-567-027
Ultrapure DNase/RNase Free Distilled Water	Fisher Scientific	10–977-015
Collagenase D	Sigma-Aldrich	11088858001

Critical commercial assays		

NEBNext Poly(A) mRNA Magnetic Isolation Module	New England Biolabs	E7490S
Nebnext Ultra II Directional RNA Library Prep Kit for Illumina - 24 rxns	New England Biolabs	E7760S
NEBNext Multiplex Oligos for Illumina (Index Primers Set 1)	New England Biolabs	E7335S
NEBNext Multiplex Oligos for Illumina (Index Primers Set 2)	New England Biolabs	E7500S
NEBNext Multiplex Oligos for Illumina (Index Primers Set 3)	New England Biolabs	E7710S
NEBNext Multiplex Oligos for Illumina (Index Primers Set 4)	New England Biolabs	E7730S
NEBNext Multiplex Oligos for Illumina (96 Index Primers) - 96 reactions	New England Biolabs	E6609S
Agencourt RNAClean XP	Beckman Coulter	A63987
RNAscope Multiplex Fluorescent Detection Reagents v2	Advanced Cell Diagnostics	323100
SuperScript™ IV First-Strand Synthesis System	Thermo Fisher Scientific	18091200
LIVE/DEAD Fixable Near-IR Dead Cell Stain Kit, for 633 or 635 nm excitation	Thermo Fisher Scientific	L10119
LIVE/DEAD™ Fixable Blue Dead Cell Stain Kit, for UV excitation	Thermo Fisher Scientific	L23105
Ultracomp eBeads™ Compensation Beads	Thermo Fisher Scientific	01–2222-42
PicoPure™ RNA Isolation Kit	Thermo Fisher Scientific	KIT0204

Deposited data		

Human intestine-, lung-draining LNs *RNAseq*	This study	GEO: GSE281776
exGF/GF mouse intestine LNs *RNAseq*	This study	GEO: GSE281824
*Rag1*^− /−^ mouse intestine LNs *RNAseq*	This study	GEO: GSE282128
Vitamin A deficient diet-fed B6 mouse intestine LNs *RNAseq*	This study	GEO: GSE281117
LN CD169 macrophages *RNAseq*	This study	GEO: GSE281209
LN sorted macrophages *RNAseq*	This study	GEO: GSE282129
Human LN non-hematopoietic cells *scRNAseq*	Abe et al.^6^	https://ega-archive.org/EGAD00001008311
Human LN lymphatic endothelial cell *scRNAseq*	Takeda et al.^7^	GEO: GSE124494
Human tonsil immune cells *scRNAseq*	De Martin and Stanossek et al.^20^	https://www.ebi.ac.uk/biostudies/E-MTAB-11715
Mouse LN immune cells *scRNAseq*	Tong and Mang et al.^18^	GEO: GSE160199
Mouse LN FRC/LEC *scRNAseq*	Lenti et al.^23^	GEO: GSE151623

Experimental models: Organisms/strains		

Mouse: B6 (C57BL/6J)	Jackson Laboratories	Strain #:000664
Mouse: *Rag1*^− /−^ (B6.129S7-Rag1^tm1Mom^/J)	Jackson Laboratories	Strain #:002216
Mouse: RiboTag (B6J.129(Cg)-Rpl22^tm1.1Psam^/SjJ)	Jackson Laboratories	Strain #:029977
Mouse: Germ Free C57BL/6 (Black 6)	Taconic Biosciences	C57BL/6NTac
Mouse: CD169-Cre, B6-Siglec1/Cre KI	Dr. Masato Tanaka, Tokyo University of Pharmacy and Life Sciences, School of Life Science	N/A

Oligonucleotides		

*36b4* Fwd: 5^′^-GCCGTGATGCCCAGGGAAGAC-3^′^ *36b4* Rev: 5^′^-CATCTGCTTGGAGCCCACGTT-3^′^	Moest et al.^57^	N/A
*Aldh1a2* Fwd: 5^′^-ATGGATGCGTCTGAAAGAGG-3^′^ *Aldh1a2* Rev: 5^′^-TGACTCCCTGCAAATCGATG-3^′^	IDT	N/A
*Gpc3* Fwd: 5^′^-AGAAACCTTATCCAGCCGAAG-3^′^ *Gpc3* Rev: 5^′^-AGTTCTTGTCCGTTCCAGC-3^′^	IDT	N/A
*Hoxc10* Fwd: 5^′^-CCCAGTCCCAATGAGATCAAG-3^′^ *Hoxc10* Rev: 5^′^-GGTGTTTTCTGCCTTTATCTCC-3^′^	IDT	N/A
*Hoxc9* Fwd: 5^′^-GCAAGCACAAAGAGGAGAAGG-3^′^ *Hoxc9* Rev: 5^′^-GCGTCTGGTACTTGGTGTAG-3^′^	IDT	N/A
*Cxcl14* Fwd: 5^′^-TGAAGCCAAAGTACCCACAC-3^′^ *Cxcl14* Rev: 5^′^-TCCAGGCATTGTACCACTTG-3^′^	IDT	N/A
*Itih4* Fwd: 5^′^- TTGCTTCTGGCTCTGACTTC −3^′^ *Itih4* Rev: 5^′^- TTCCTCTGGTATGGTGCTTTC −3^′^	IDT	N/A
*Mmp3* Fwd: 5^′^- GATGAACGATGGACAGAGGATG −3^′^ *Mmp3* Rev: 5^′^- AAACGGGACAAGTCTGTGG −3^′^	IDT	N/A
*Ccl19* Fwd: 5^′^- ATGTGAATCACTCTGGCCCAGGAA −3^′^ *Ccl19* Rev: 5^′^- AAGCGGCTTTATTGGAAGCTCTGC −3^′^	IDT	N/A
*Clec4g* Fwd: 5^′^- AGTGCAATTGAAGAGGTCTCC −3^′^ *Clec4g* Rev: 5^′^- GACAGTAGGGTGCTCAGAATG −3^′^	IDT	N/A
*Il22ra2* Fwd: 5^′^- CCAGAAGGTCCGATTTCAGTC −3^′^ *Il22ra2* Rev: 5^′^- GCAGTCAACTTTATCTTCCCATTG −3^′^	IDT	N/A
*Aox3* Fwd: 5^′^- CCACAGGAGAGGCAGTATTTTG −3^′^ *Aox3* Rev: 5^′^- ACCACATCAACCACACCAAG −3^′^	IDT	N/A
*Gbp2b* Fwd: 5^′^- CGAGAAGCCAGAACATACCC −3^′^ *Gbp2b* Rev: 5^′^- TGGTTGATGGTTCCTATGCTG −3^′^	IDT	N/A
*C6* Fwd: 5^′^- CCTCCAAGCTCTGCAAAATTG −3^′^ *C6* Rev: 5^′^- CCTCCCACAGTTCCTTTCATC −3^′^	IDT	N/A
*Lum* Fwd: 5’ -TAAGCTTAAGAGTATACCAACAGTTAATG −3^′^ *Lum* Rev: 5^′^- GCGCAGATGCTTGATCTTG −3^′^	IDT	N/A
*P2rx2* Fwd: 5^′^- GGACGCTGTGTACCCTATTAC −3^′^ *P2rx2* Rev: 5^′^- GGGATAGTGGATGCTGTTCTTG −3^′^	IDT	N/A
*Kcnk3* Fwd: 5^′^- CTCCTTCTACTTCGCCATCAC −3^′^ *Kcnk3* Rev: 5^′^- GGCTCTGGAACATGACTAGTG −3^′^	IDT	N/A
*Ppp1r9a* Fwd: 5^′^- TGTTTTCCCCATCTGACCTG −3^′^ *Ppp1r9a* Rev: 5^′^- CCCACCTTACTTTCTCATTTTCG −3^′^	IDT	N/A
*Hk3* Fwd: 5^′^- ATGATCCCCGTTGTGAGATG −3^′^ *Hk3* Rev: 5^′^- ACCCCACTCCATGTTGATG −3^′^	IDT	N/A
*Rspo3* Fwd: 5^′^- CAACCAGCGAGACAAGAACT −3^′^ *Rspo3* Rev: 5^′^- TCCAAACCTTTGCTGTCAGAG −3^′^	IDT	N/A
*Wfdc1* Fwd: 5^′^- CCTCGGGATGGGTAACTG −3^′^ *Wfdc1* Rev: 5^′^- TGTCGCTGCAACCTCTTC −3^′^	IDT	N/A
*Msr1* Fwd: 5^′^- GGGAACACTCACAGACACTG −3^′^ *Msr1* Rev: 5^′^- CCCGATCACCTTTAACACCTG −3^′^	IDT	N/A
*Acta2 (*NM_007392.3, target region 444–543) AACCCTAAGGCCAACCGGGAGAAAATGAC CCAGATTATGTTTGAGACCTTCAATGTCCC CGCCATGTATGTGGCTATTCAGGCTGTGC TGTCCCTCTATG	NanoString	N/A
*Actb (*NM_007393.1, target region 816–915) CAGGTCATCACTATTGGCAACGAGCGGT TCCGATGCCCTGAGGCTCTTTTCCAGCC TTCCTTCTTGGGTATGGAATCCTGTGGC ATCCATGAAACTACAT	NanoString	N/A
*Aldh1a2 (*NM_009022.3, target region 395–494) CTGCAAGCTTTTTACATCGATTTGCAGGGAG TCATCAAAACCCTGAGGTATTATGCAGGCTG GGCTGATAAAATTCACGGAATGACCATTCC TGTAGATG	NanoString	N/A
*Aox3 (*NM_023617.2, target region 1766–1865) AACTCCTACACATTCTGGAAGACTTCCCTT TAACCATGCCTTATGGGATGCAGTCATTT CAGGATGTAGACTTCCAACAGCCTCTG CAAGACCCAATCGG	NanoString	N/A
*C6 (*NM_016704.2, target region 171–270) CTGATTGACAAGAGTGAAGCCTGTTTC TGTGACCACTACCCATGGACTCACTG GTCCAGCTGTTCTAAGTCCTGCAAT TCTGGAACCCAGAGCAGACAGA	NanoString	N/A
*Ccl19 (*NM_011888.2, target region 466–565) CTTCTGCCAAGAACAAAGGCAACAGCACC AGAAGGAGCCCTGTGTCTTGAGTAAAGAG ATGTGAATCACTCTGGCCCAGGAAACCA AGGACCAGAAGAGA	NanoString	N/A
*Cd34 (*NM_001111059.1, target region 561–660) CTGATTATTCGCCTAATAATAGCAGCTTTGAG ATGACATCACCCACCGAGCCATATGCTTAC ACATCATCTTCTGCTCCGAGTGCCATT AAGGGAGAAAT	NanoString	N/A
*Clec4g (*NM_029465.3, target region 649–748) CACACACGTGGCCGGGGCTATTGGTTGGG TCTGAGGGCAGTTCGCCACCTCAACAAGA TTCAAGGCTACCGGTGGGTAGATGGAG CCTCACTCAACTTCA	NanoString	N/A
*Cr2 (*NM_007758.2, target region 1651–1750) TGGTTTATAGAAATCCGTCTTTGTAAAGAA ATCACCTGCCCACCACCTCCTGTTATACA CAACGGGACACATACATGGAGTTCCTCA GAAGATGTCCCAT	NanoString	N/A
*Cxcl14 (*XM_006517307.1, target region 447–546) GTGGACGGGTCCAAGTGTAAGTGTTCCCGGAA GGGGCCCAAGATCCGCTACAGCGACGTGAAG AAGCTGGAAATGAAGCCAAAGTACCCACACT GCGAGG	NanoString	N/A
*Cxcl9 (*NM_008599.4, target region 113–212) TCGAGGAACCCTAGTGATAAGGAATGCA CGATGCTCCTGCATCAGCACCAGCCGA GGCACGATCCACTACAAATCCCTCAAA GACCTCAAACAGTTTGCC	NanoString	N/A
*Flt4 (*NM_008029.3, target region 5691–5790) CTGTGTCACCTCTCCTGACTTGTAACTACA CACATACGAAATGTACTGGGGAAGAAAAC GTGCTCTGCCCTTTTTGGCAGTGTTCTGA AATGAGCTAAGA	NanoString	N/A
*Gbp2b (*NM_010259.2, target region 167–266) ATCCTGTCTGCTATCCAAAATCCTGTGGTG GTCGTAGCAATAGTGGGCTTCTACCACAC AGGCAAATCCTATCTGATGAATAAGCTGG CTGGAAAGCAGA	NanoString	N/A
*Gpc3 (*NM_016697.3, target region 901–1000) ACCTCAAGTTTAGTAAGGACTGTGGCCGTA TGCTCACCCGAATGTGGTATTGCTCTTACT GCCAGGGACTGATGATGGTTAAGCCTTG CGGTGGTTATTG	NanoString	N/A
*Hk3 (*NM_001033245.4, target region 2545–2644) CATATCCTCCTGCACTTAACCAATCTCGGAGT TCTCTTCCGAGGCCAGAAGACTCAATGCCTT CAGGCCAGGGACATCTTCAAGACTAAGTT CCTCTCTG	NanoString	N/A
*Hoxc10 (*NM_010462.5, target region 1351–1450) GTGTGTGTGTCAACTCTTCAGTCACCCATGCA CTCACATACAGCATTCTGTTCTCCATGCAAAG TTGAGGTCAAATGCACCCGATTAGAGGGGAA AGAAA	NanoString	N/A
*Hoxc9 (*NM_008272.3, target region 1567–1666) GAAGGGGAAAAATGCAAACTCTTGCGATGT GGGAGGGTTAAGTGTTGAGAAATTGGTGTT TAGAGTTAGTTCTATCCATCGAGGAGGAG GCAGGAGAGAA	NanoString	N/A
*Il22ra2 (*NM_178258.5, target region 363–462) CCGCTTCACTCCATGGTGGGAAACAAAAC TAGATCCTCCGGTCGTGACTATAACCCG AGTTAACGCATCTTTGCGGGTGCTTCTC CGTCCTCCAGAGTTG	NanoString	N/A
*Inmt (*NM_009349.3, target region 821–920) GCCTTCCCTGGGAAAGTTATAGCAATCA TTTGTAAAGCCTTGCTCCACAGCTTTCT AACACGGACCATGTTGCTTCTATCTAAT ACCTGGTCACAGTCAC	NanoString	N/A
*Itih4 (*NM_001159299.1, target region 2676–2775)	NanoString	N/A
ACAACGTTAAAGGGGAGCTTGGTCAGTTTTAC		
CGGGACATCGTCTGGGAGCCACCCGTCGA		
GCCAGATAATACAAAACGGACAGTCAAAG		
TTCAAGGAGT		
*Kcnk3 (*NM_010608.2, target region 681–780) CTCCTACTACGAGCGCTGGACTTTCTTCC AGGCCTATTACTACTGCTTCATCACCCTC ACCACCATCGGCTTCGGCGACTATGTG GCGCTGCAGAAGGAC	NanoString	N/A
*Lum (*NM_008524.2, target region 871–970) CGACGGGCTGGTCAACTTGACCTTCATTT ATCTTCAACACAACCAGCTCAAAGAGGAT GCTGTCTCGGCTTCTCTGAAAGGTCTCA AATCACTAGAGTAC	NanoString	N/A
*Mmp3 (*NM_010809.1, target region 1576–1675) TCTTTGTGAAAGGAAGTGCTTTGTTCAGCATG TGCTATGGCAGAACCAAACAGGAGCTATGG ATGACACCAGTCAACGTCAAGTTGTCAAA GGATGTTCA	NanoString	N/A
*Msr1 (*NM_001113326.1, target region 556–655) GATTTCGTCAGTCCAGGAACATGGGAATTCA CTGGATGCAATCTCCAAGTCCTTGCAGAGT CTGAATATGACACTGCTTGATGTTCAACTC CATACAGAA	NanoString	N/A
*Nr4a1 (*NM_010444.1, target region 1316–1415) ATGCCGGTGACGTGCAACAATTTTATGACTT GCTCTCTGGTTCCCTGGACGTTATCCGAAA GTGGGCAGAAAAAATCCCTGGCTTCATT GAGCTTTGCCC	NanoString	N/A
*P2rx2 (*NM_001164833.1, target region 1435–1534) CGTGACTGGGAAACAGACACCTGTGCAAGAAG ATAGGCATCTTGCTCTGGGCCAGGCTTACATT CTTCCTCTCCCTAAGGCTTCTGGGGAGAAGTGGGTC	NanoString	N/A
*Polr1b (*NM_009086.2, target region 2796–2895) TGCCTTTCACTGAGAGTGGCATGATGCCGGA CATTCTGTTTAATCCTCACGGGTTTCCCTCC CGTATGACCATAGGTATGTTAATCGAGAG CATGGCTGG	NanoString	N/A
*Ppp1r9a (*NM_181595.3, target region 503–602) CCAGCAGAGCAGAGGTAGAAAGTATGGCTC CAATGTCAACAGAATTAAAAATCTATTTATGC AGATGGGTATGGAACCCAGCGAGAATGCT GCTATCATT	NanoString	N/A
*Prox1 (*NM_008937.2, target region 2113–2212) AGCACCGCAGAAGGACTCTCTTTGTCACTC ATAAAGTCTGAGTGTGGAGATCTTCAAGAT ATGTCCGACATCTCACCTTATTCAGGAA GCGCAATGCAGG	NanoString	N/A
*Rpl19 (*NM_009078.1, target region 21–120) GAAGAGGCTTGCCTCTAGTGTCCTCCGC TGCGGGAAAAAGAAGGTCTGGTTGGATC CCAATGAGACCAATGAAATCGCCAATGC CAACTCCCGTCAGCAG	NanoString	N/A
*Rplp0 (*NM_007475.5, target region 496–595) TCAGAACACTGGTCTAGGACCCGAGAAGA CCTCCTTCTTCCAGGCTTTGGGCATCACC ACGAAAATCTCCAGAGGCACCATTGAAA TTCTGAGTGATGTG	NanoString	N/A
*Rspo3 (*NM_028351.3, target region 2019–2118) CTGGAGACCATCTTGTGCTTTCCAGAACCG TGAGGGGTTTTGGTCACCTGGAACAGGGCT CCAATCTATATTAGCACTGTGTGGTTGAT CTTCCACTACT	NanoString	N/A
*Tbp (*NM_013684.3, target region 71–170) GTGGCGGGTATCTGCTGGCGGTTTGG CTAGGTTTCTGCGGTCGCGTCATTTT CTCCGCAGTGCCCAGCATCACTATT TCATGGTGTGTGAAGATAACCCA	NanoString	N/A
*Tnfsf11 (*NM_011613.3, target region 616–715) CCAGCGAGGCAAGCCTGAGGCCCAGCCAT TTGCACACCTCACCATCAATGCTGCCAGCA TCCCATCGGGTTCCCATAAAGTCACTCT GTCCTCTTGGTAC	NanoString	N/A
*Wfdc1 (*NM_023395.2, target region 773–872) GGCAGAGCCTGGGAAGGGACAACAGAG GCACTTTCCATAAAGTGAAGGCTGGCTG CCTTTGCGGGGCCTTTCCTGTGTCTTC CACACGCTAAGCCTTGGA	NanoString	N/A

Software and algorithms		

QuPath v0.5.1		https://qupath.github.io/
GraphPad Prism v10.1.1	GraphPad	www.graphpad.com
Adobe Illustrator v27.0	Adobe	www.adobe.com/products/illustrator
R (v4.3.2)	The R Foundation	https://www.r-project.org/
RStudio v4.3.2	RStudio, Inc.	www.rstudio.com
ImageJ v1.53a	–	N/A
FlowJo (v10.8.1)	Tree Star	https://www.flowjo.com/
nSolver Analysis Software 4.0	NanoString Technologies	https://nanostring.com/

Other		

RNAscope probe: *Inmt* (NM_009349.3, target region 3–1017 bp)	Advanced Cell Diagnostics	486371-C2
RNAscope probe: *Lum* (NM_008524.2, target region 254–1336 bp)	Advanced Cell Diagnostics	480361-C3
RNAscope probe: *Hk3* (NM_001033245.4, target region 361–1165 bp)	Advanced Cell Diagnostics	1055121-C2
RNAscope probe: *Clec4g* (NM_029465.3, target region 257–1176 bp)	Advanced Cell Diagnostics	503371-C2
Vitamin A deficient diet	TD.86143	Inotiv
Vitamin A sufficient diet (with vitamin A added back at 20 IU/g	TD.91280	Inotiv
No fat diet (0.5% of calories from fat, which come from the grain shells)	TD.180890	Inotiv
CHOW diet (control for no fat diet, 16% of calories from fat)	2020×	Inotiv
Dapi Fluoromount G Clear Mounting Media	Thermo Fisher Scientific	OB010020

## Highlights

- Gut lymph nodes differ most in their stromal cell composition
- This is true in humans and mice
- This difference is induced by vitamin A and amplified by the microbiota
