# Supplementary material for: Medullary stromal cells define small intestinal lymph node identity in humans and mice
Source: Cell Rep. Author manuscript; Available in PMC 2026 Apr 6. (PMC13051315; doi:10.1016/j.celrep.2025.116441)
Supplement: 1 [file NIHMS2120127-supplement-1.pdf]

Cell Reports, Volume *44*

Supplemental information

Medullary stromal cells define small intestinal lymph node identity in humans and mice

Aliia R. Fatkhullina, Johnathan Kent, Hailey Brown, Nathaniel Christiansen, Wioletta Lisicka, Maria Lucia Madariaga, and Daria Esterházy

Supplemental Information

Medullary stromal cells define small intestinal lymph node identity in humans and mice

Aliia R. Fatkhullina, Johnathan Kent, Hailey Brown, Nathaniel Christiansen, Wioletta Lisicka, Maria Lucia Madariaga and Daria Esterházy

Figure S1

A

Donor number	Age	Sex	Race	Smoking	BMI	Cause of Death	PMHx
1	54	M	Caucasian	Non Smoker	32.6	Overdose	Opiate abuse disorder
2	74	M	Black	Non Smoker	28.6	Intracranial hemorrhage	Atrial fibrillation, COPD, HTN, HLD, CHF, DM, stroke, CKD stage 3, anemia
3	50	F	Hispanic	Smoking	29.5	Stroke	HTN
4	42	F	Asian	Non Smoker	35.6	Stroke	DM
5	42	M	Hispanic	Smoker	26.1	Overose	Opiate abuse disorder, Unknown
6	72	M	Caucasian	Non Smoker	25.3	Intracranial hemorrhage	HTN, Hypothyroidism, Atrial Fibrillation, stroke
7	35	F	Caucasian	Smoker	21.0	Suicide	Absence seizures, migraines, hydrocephalus, kidney stone, ADHD
8	46	F	Black	Unknown	36.7	Overdose	Cocaine Use, Unknown
9	41	F	Hispanic	Smoker	34.8	Overdose	

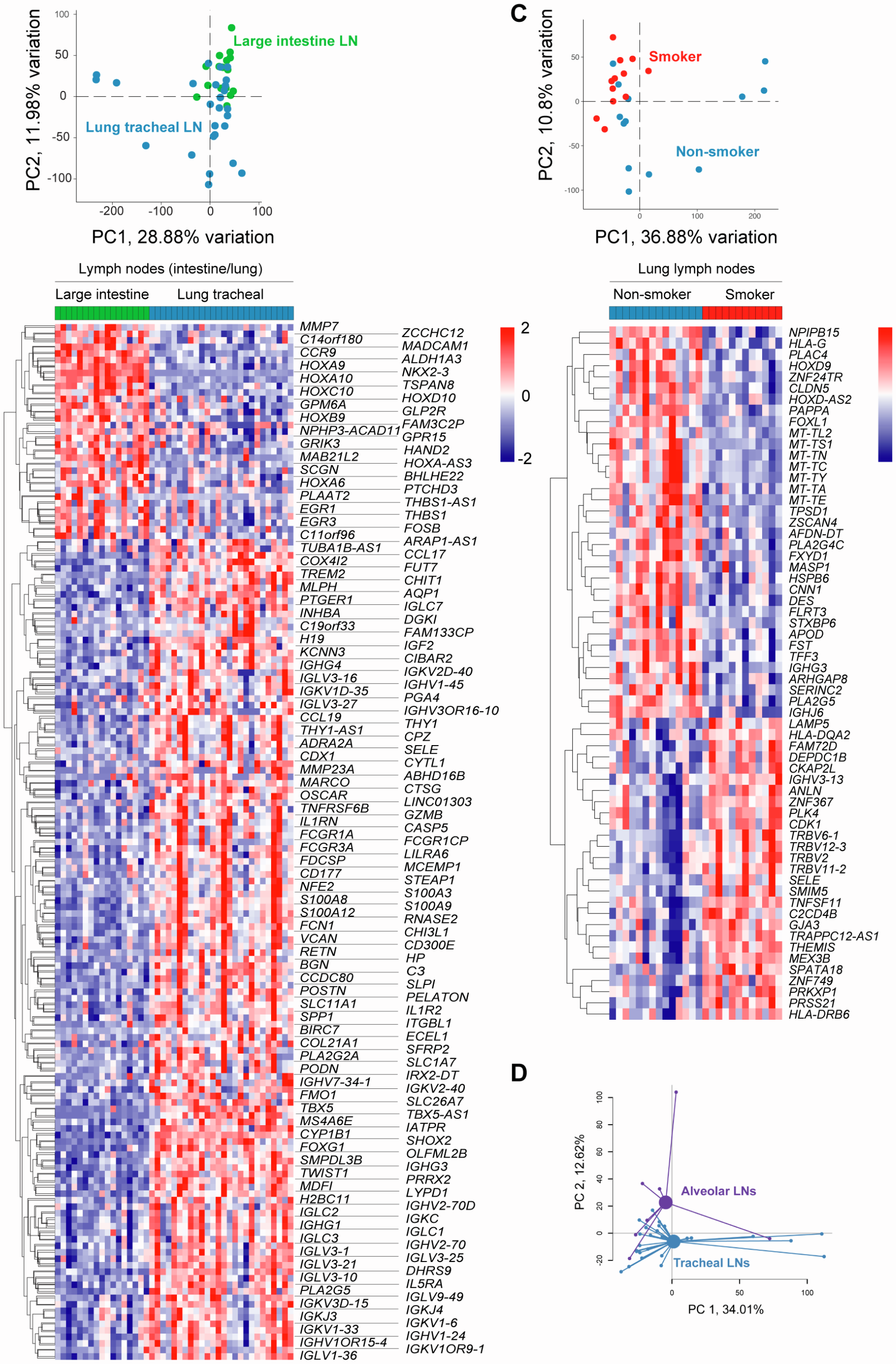

Figure S1. Human mucosal LNs are transcriptionally distinct according to the organ or gut region they drain. **A**. Table showing demographics of LN donors used for *RNAseq*. **B**, **C**. Principal component plot and heatmap and hierarchical clustering of all DEGs with 1.5 logFC of all large intestinal and all lung LNs (B) or all lung LNs color-coded by smoking history (C). **D.** Principal component plot of tracheal *versus* alveolar LNs.

Figure S2

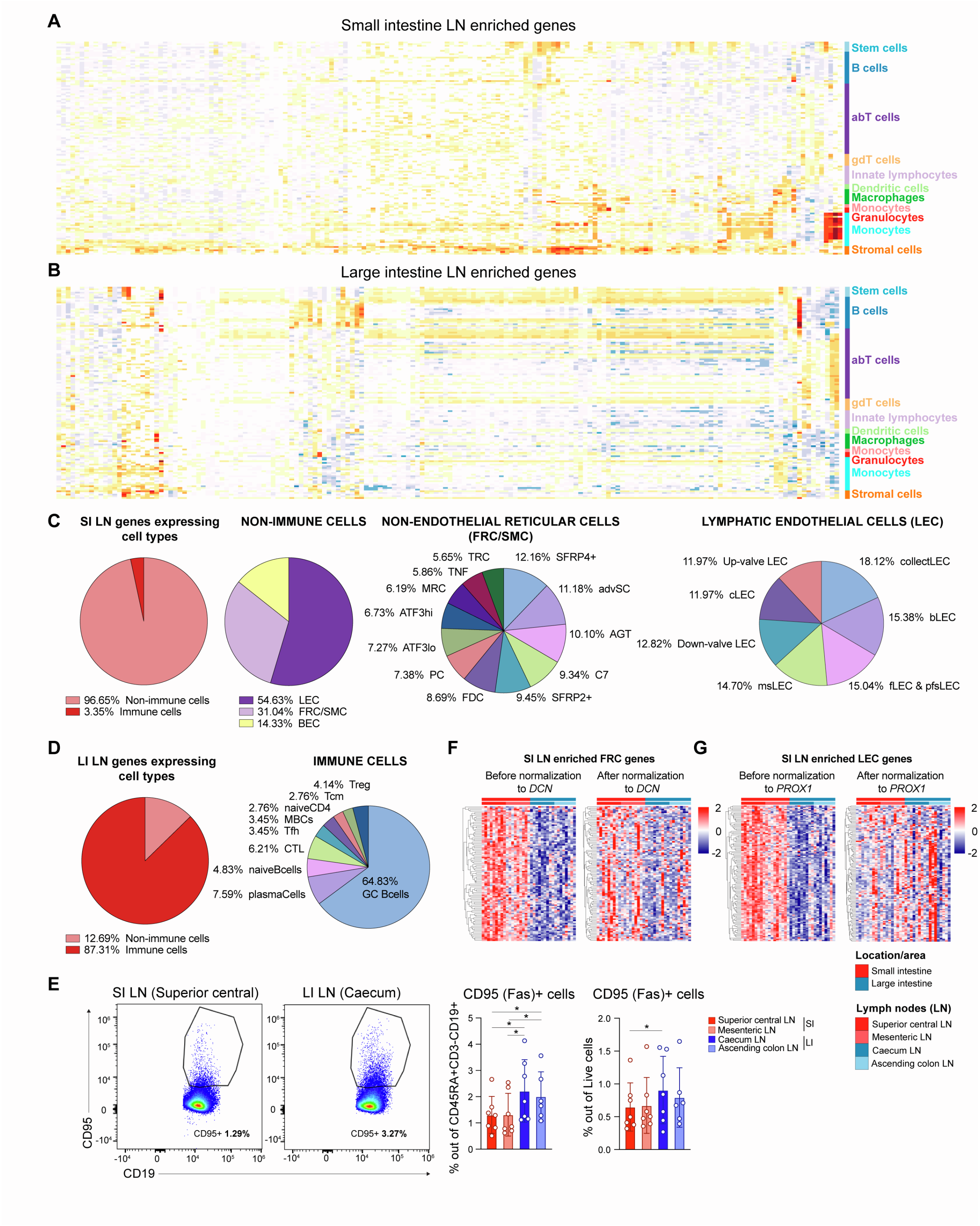

A
Small intestine LN enriched genes
Stem cells
B cells
abT cells
gdT cells
Innate lymphocytes
Dendritic cells
Macrophages
Monocytes
Granulocytes
Monocytes
Stromal cells
B
Large intestine LN enriched genes
Stem cells
B cells
abT cells
gdT cells
Innate lymphocytes
Dendritic cells
Macrophages
Monocytes
Granulocytes
Monocytes
Stromal cells
C
SI LN genes expressing cell types
96.65% Non-immune cells
3.35% Immune cells
NON-IMMUNE CELLS
54.63% LEC
31.04% FRC/SMC
14.33% BEC
NON-ENDOTHELIAL RETICULAR CELLS (FRC/SMC)
5.65% TRC
12.16% SFRP4+
5.86% TNF
6.19% MRC
11.18% advSC
6.73% ATF3hi
10.10% AGT
7.27% ATF3lo
7.38% PC
9.34% C7
8.69% FDC
9.45% SFRP2+
LYMPHATIC ENDOTHELIAL CELLS (LEC)
11.97% Up-valve LEC
18.12% collectLEC
11.97% cLEC
15.38% bLEC
12.82% Down-valve LEC
14.70% msLEC
15.04% fLEC & pfsLEC
D
LI LN genes expressing cell types
12.69% Non-immune cells
87.31% Immune cells
IMMUNE CELLS
4.14% Treg
2.76% Tcm
2.76% naiveCD4
3.45% MBCs
3.45% Tfh
6.21% CTL
4.83% naiveBcells
7.59% plasmaCells
64.83% GC Bcells
F
SI LN enriched FRC genes
Before normalization to DCN
After normalization to DCN
2
0
-2
G
SI LN enriched LEC genes
Before normalization to PROX1
After normalization to PROX1
2
0
-2
Location/area
Small intestine
Large intestine
Lymph nodes (LN)
Superior central LN
Mesenteric LN
Caecum LN
Ascending colon LN
E
SI LN (Superior central)
LI LN (Caecum)
CD95
CD19
CD95+ 1.29%
CD95+ 3.27%
CD95 (Fas)+ cells
% out of CD45RA+CD3-CD19+
CD95 (Fas)+ cells
% out of Live cells
Superior central LN
Mesenteric LN
Caecum LN
Ascending colon LN
SI
LI

Figure S2. Human small intestinal LN enriched transcripts primarily map to medullary sinus stromal cells, while those of large intestinal LNs to germinal center B cells. A, **B**. Heat map of expression of top 150 DEGs enriched in SI LNs (A) or LI LNs (B) within human immune cell and stromal cell populations in Immgen *RNAseq* database. **C**. Contribution of immune *versus* non-immune cells and non-immune subpopulations to SI LN enriched transcripts. **D**. Contribution of immune *versus* non-immune cells and immune subpopulations to LI LN enriched transcripts. **E.** Representative flow cytometry plots and average percentages of LN GC B cell subpopulations in the human superior central, mesenteric, caecal, and ascending colon LNs out of total $CD19^+$ B cells (left) or total live cells (right) (*n*=6-7, as indicated by dots). **F-G**. Heat maps and hierarchical clustering of SI-enriched FRC (F) or LEC (G) transcripts before and after normalization to FRC marker *DCN* or LEC marker *PROX1* expression. Data are represented as mean +/- SEM, * $p<0.05$ by paired t-test.

Figure S3

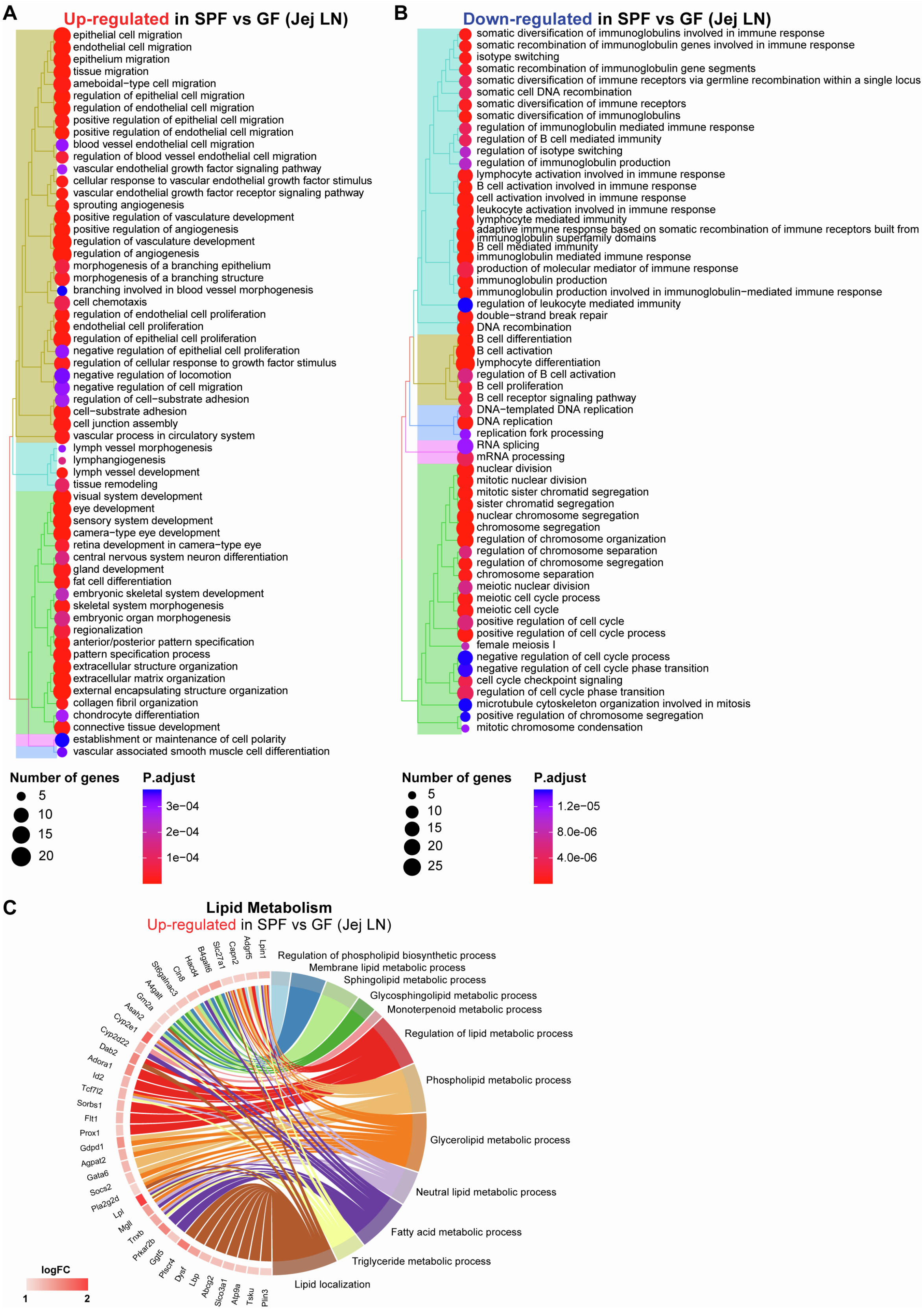

A
Up-regulated in SPF vs GF (Jej LN)
epithelial cell migration
endothelial cell migration
epithelium migration
tissue migration
ameboidal−type cell migration
regulation of epithelial cell migration
regulation of endothelial cell migration
positive regulation of epithelial cell migration
positive regulation of endothelial cell migration
blood vessel endothelial cell migration
regulation of blood vessel endothelial cell migration
vascular endothelial growth factor signaling pathway
cellular response to vascular endothelial growth factor stimulus
vascular endothelial growth factor receptor signaling pathway
sprouting angiogenesis
positive regulation of vasculature development
positive regulation of angiogenesis
regulation of vasculature development
regulation of angiogenesis
morphogenesis of a branching epithelium
morphogenesis of a branching structure
branching involved in blood vessel morphogenesis
cell chemotaxis
regulation of endothelial cell proliferation
endothelial cell proliferation
regulation of epithelial cell proliferation
negative regulation of epithelial cell proliferation
regulation of cellular response to growth factor stimulus
negative regulation of locomotion
negative regulation of cell migration
regulation of cell−substrate adhesion
cell−substrate adhesion
cell junction assembly
vascular process in circulatory system
lymph vessel morphogenesis
lymphangiogenesis
lymph vessel development
tissue remodeling
visual system development
eye development
sensory system development
camera−type eye development
retina development in camera−type eye
central nervous system neuron differentiation
gland development
fat cell differentiation
embryonic skeletal system development
skeletal system morphogenesis
embryonic organ morphogenesis
regionalization
anterior/posterior pattern specification
pattern specification process
extracellular structure organization
extracellular matrix organization
external encapsulating structure organization
collagen fibril organization
chondrocyte differentiation
connective tissue development
establishment or maintenance of cell polarity
vascular associated smooth muscle cell differentiation
Number of genes
5
10
15
20
P.adjust
3e−04
2e−04
1e−04
B
Down-regulated in SPF vs GF (Jej LN)
somatic diversification of immunoglobulins involved in immune response
somatic recombination of immunoglobulin genes involved in immune response
isotype switching
somatic recombination of immunoglobulin gene segments
somatic diversification of immune receptors via germline recombination within a single locus
somatic cell DNA recombination
somatic diversification of immune receptors
somatic diversification of immunoglobulins
regulation of immunoglobulin mediated immune response
regulation of B cell mediated immunity
regulation of isotype switching
regulation of immunoglobulin production
lymphocyte activation involved in immune response
B cell activation involved in immune response
cell activation involved in immune response
leukocyte activation involved in immune response
lymphocyte mediated immunity
adaptive immune response based on somatic recombination of immune receptors built from immunoglobulin superfamily domains
B cell mediated immunity
immunoglobulin mediated immune response
production of molecular mediator of immune response
immunoglobulin production
immunoglobulin production involved in immunoglobulin−mediated immune response
regulation of leukocyte mediated immunity
double−strand break repair
DNA recombination
B cell differentiation
B cell activation
lymphocyte differentiation
regulation of B cell activation
B cell proliferation
B cell receptor signaling pathway
DNA−templated DNA replication
DNA replication
replication fork processing
RNA splicing
mRNA processing
nuclear division
mitotic nuclear division
mitotic sister chromatid segregation
sister chromatid segregation
nuclear chromosome segregation
chromosome segregation
regulation of chromosome organization
regulation of chromosome separation
regulation of chromosome segregation
chromosome separation
meiotic nuclear division
meiotic cell cycle process
meiotic cell cycle
positive regulation of cell cycle
positive regulation of cell cycle process
female meiosis I
negative regulation of cell cycle process
negative regulation of cell cycle phase transition
cell cycle checkpoint signaling
regulation of cell cycle phase transition
microtubule cytoskeleton organization involved in mitosis
positive regulation of chromosome segregation
mitotic chromosome condensation
Number of genes
5
10
15
20
25
P.adjust
1.2e−05
8.0e−06
4.0e−06
C
Lipid Metabolism
Up-regulated in SPF vs GF (Jej LN)
Regulation of phospholipid biosynthetic process
Membrane lipid metabolic process
Sphingolipid metabolic process
Glycosphingolipid metabolic process
Monoterpenoid metabolic process
Regulation of lipid metabolic process
Phospholipid metabolic process
Glycerolipid metabolic process
Neutral lipid metabolic process
Fatty acid metabolic process
Triglyceride metabolic process
Lipid localization
Lpin1
Adgrf5
Capn2
Slc27a1
B4galt6
Hacd4
Cln8
St6galnac3
A4galt
Gm2a
Asah2
Cyp2e1
Cyp2d22
Dab2
Adora1
Id2
Tcf7l2
Sorbs1
Flt1
Prox1
Gdpd1
Agpat2
Gata6
Socs2
Pla2g2d
Lpl
Mgll
Tnxb
Prkar2b
Ggt5
Plscr4
Dysf
Lbp
Abcg2
Slco3a1
Atp9a
Tsku
Plin3
logFC
1
2

Figure S3. The mouse small intestinal LNs are transcriptionally distinct from those draining colon and skin, and the difference is dampened but not lost in the absence of a microbiota. **A**, **B**. Gene set enrichment analysis of pathways overrepresented (A) or downregulated (B) in jejunal LNs from SPF compared to G mice as determined by *RNAseq*. **C**. Chord diagram connecting lipid metabolic pathways and DEGs contributing to them that are enriched in jejunal LNs from SPF compared to GF mice.

Figure S4

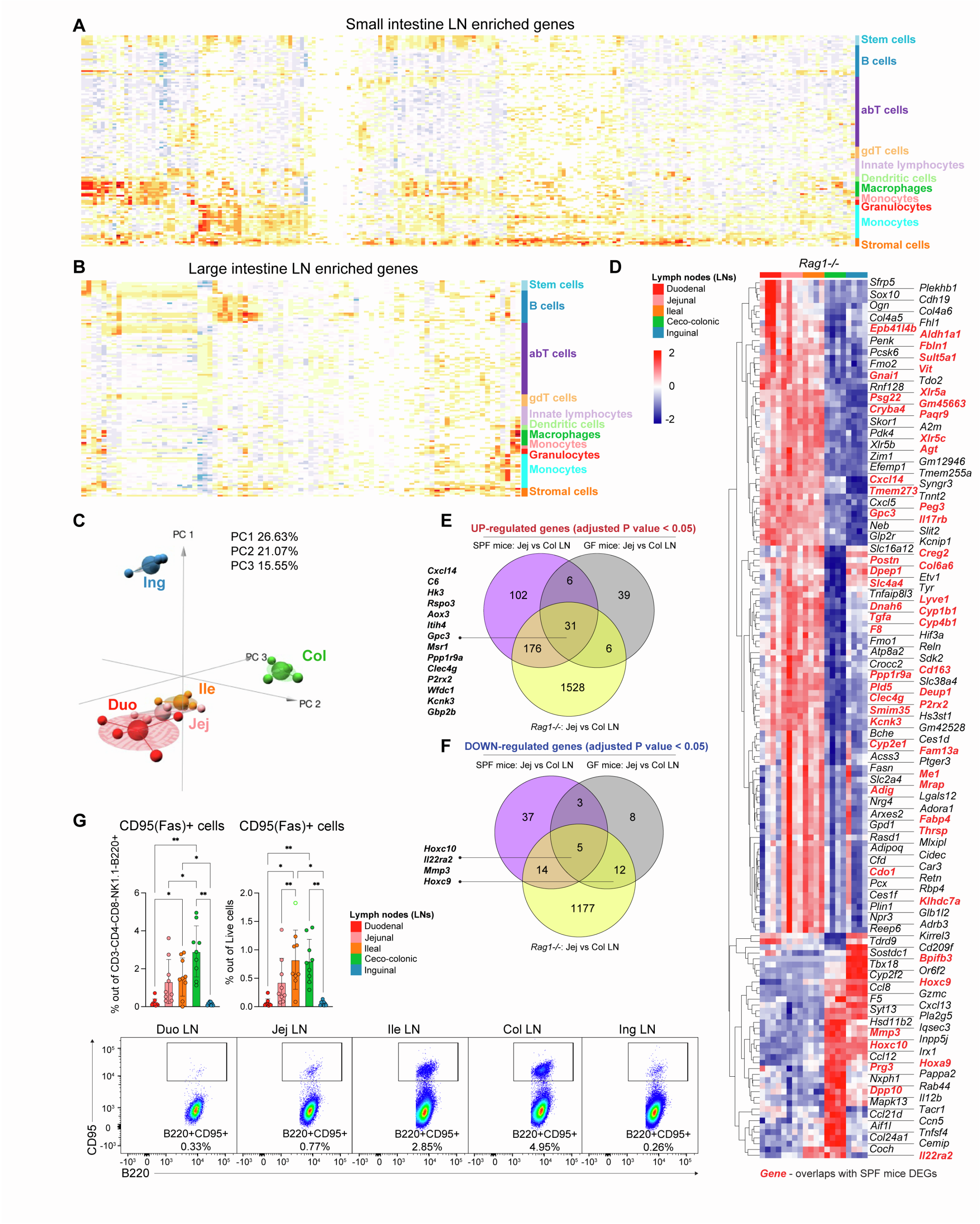

A
Small intestine LN enriched genes
Stem cells
B cells
abT cells
gdT cells
Innate lymphocytes
Dendritic cells
Macrophages
Monocytes
Granulocytes
Monocytes
Stromal cells
B
Large intestine LN enriched genes
Stem cells
B cells
abT cells
gdT cells
Innate lymphocytes
Dendritic cells
Macrophages
Monocytes
Granulocytes
Monocytes
Stromal cells
C
PC 1
PC1 26.63%
PC2 21.07%
PC3 15.55%
Ing
Col
PC 3
PC 2
Ile
Duo
Jej
D
Rag1-/-
Lymph nodes (LNs)
Duodenal
Jejunal
Ileal
Ceco-colonic
Inguinal
2
0
-2
Sfrp5
Sox10
Ogn
Col4a5
Epb41l4b
Penk
Pcsk6
Fmo2
Gnai1
Rnf128
Psg22
Cryba4
Skor1
Pdk4
Xlr5b
Zim1
Efemp1
Cxcl14
Tmem273
Cxcl5
Gpc3
Neb
Glp2r
Slc16a12
Postn
Dpep1
Slc4a4
Tnfaip8l3
Dnah6
Tgfa
F8
Fmo1
Atp8a2
Crocc2
Ppp1r9a
Pld5
Clec4g
Smim35
Kcnk3
Bche
Cyp2e1
Acss3
Fasn
Slc2a4
Adig
Nrg4
Arxes2
Gpd1
Rasd1
Adipoq
Cfd
Cdo1
Pcx
Ces1f
Plin1
Npr3
Reep6
Tdrd9
Sostdc1
Tbx18
Cyp2f2
Ccl8
F5
Syt13
Hsd11b2
Mmp3
Hoxc10
Ccl12
Prg3
Nxph1
Dpp10
Mapk13
Ccl21d
Aif1l
Col24a1
Coch
Plekhb1
Cdh19
Col4a6
Fhl1
Aldh1a1
Fbln1
Sult5a1
Vit
Tdo2
Xlr5a
Gm45663
Paqr9
A2m
Xlr5c
Agt
Gm12946
Tmem255a
Syngr3
Tnnt2
Peg3
Il17rb
Slit2
Kcnip1
Creg2
Col6a6
Etv1
Tyr
Lyve1
Cyp1b1
Cyp4b1
Hif3a
Reln
Sdk2
Cd163
Slc38a4
Deup1
P2rx2
Hs3st1
Gm42528
Ces1d
Fam13a
Ptger3
Me1
Mrap
Lgals12
Adora1
Fabp4
Thrsp
Mlxipl
Cidec
Car3
Retn
Rbp4
Klhdc7a
Glb1l2
Adrb3
Kirrel3
Cd209f
Bpifb3
Or6f2
Hoxc9
Gzmc
Cxcl13
Pla2g5
Iqsec3
Inpp5j
Irx1
Hoxa9
Pappa2
Rab44
Il12b
Tacr1
Ccn5
Tnfsf4
Cemip
Il22ra2
Gene - overlaps with SPF mice DEGs
E
UP-regulated genes (adjusted P value < 0.05)
SPF mice: Jej vs Col LN
GF mice: Jej vs Col LN
Rag1-/-: Jej vs Col LN
102
6
39
31
176
6
1528
Cxcl14
C6
Hk3
Rspo3
Aox3
Itih4
Gpc3
Msr1
Ppp1r9a
Clec4g
P2rx2
Wfdc1
Kcnk3
Gbp2b
F
DOWN-regulated genes (adjusted P value < 0.05)
SPF mice: Jej vs Col LN
GF mice: Jej vs Col LN
Rag1-/-: Jej vs Col LN
37
3
8
5
14
12
1177
Hoxc10
Il22ra2
Mmp3
Hoxc9
G
CD95(Fas)+ cells
CD95(Fas)+ cells
% out of CD3-CD4-CD8-NK1.1-B220+
% out of Live cells
Lymph nodes (LNs)
Duodenal
Jejunal
Ileal
Ceco-colonic
Inguinal
Duo LN
Jej LN
Ile LN
Col LN
Ing LN
B220+CD95+
0.33%
B220+CD95+
0.77%
B220+CD95+
2.85%
B220+CD95+
4.95%
B220+CD95+
0.26%
CD95
B220

S4. Murine small intestinal LN enriched transcripts primarily map to lymph node sinus cells, while those of large intestinal LNs to B cells. A, **B**. Heat map of expression of top 150 DEGs enriched in SI LNs (A) or LI LNs (B) within murine immune cell and stromal cell populations in Immgen *RNAseq* database. **C**. Principal component plot of duodenal (duo), jejunal (jej), ileal (ile), ceco-colonic (col), and inguinal (ing) LNs from *Rag1*-/- mice, *n*=4 per LN. **D**. Heat map and hierarchical clustering of the top ~150 DEGs (logFC > 1.45, adjusted P value < 0.05) between jejunal and ceco-colonic LNs of *Rag1*-/- mice across all LNs on which *RNAseq* was performed. Symbols of transcripts that are also DE between WT (SPF)/GF SI vs LI LNs are in red. **E, F.** Venn diagram showing the overlap between SI (jejunal, E) and LI (colon, F) LN DEGs of SPF, GF, and *Rag1*-/- mice. **G.** Representative flow cytometry plots (A) and average percentages (B) of LN GC B cells subpopulations in duodenal (Duo), jejunal (Jej), ileal (Ile), ceco-colonic (Col), and inguinal (Ing) LNs of 8-week-old C57BL/6 mice out of B220 (left) or live cells (right) (*n*=9). Data was pooled from two independent experiments. Data are represented as mean +/- SEM, * p<0.05, ** p<0.01 by RM one-way ANOVA test.

Figure S5

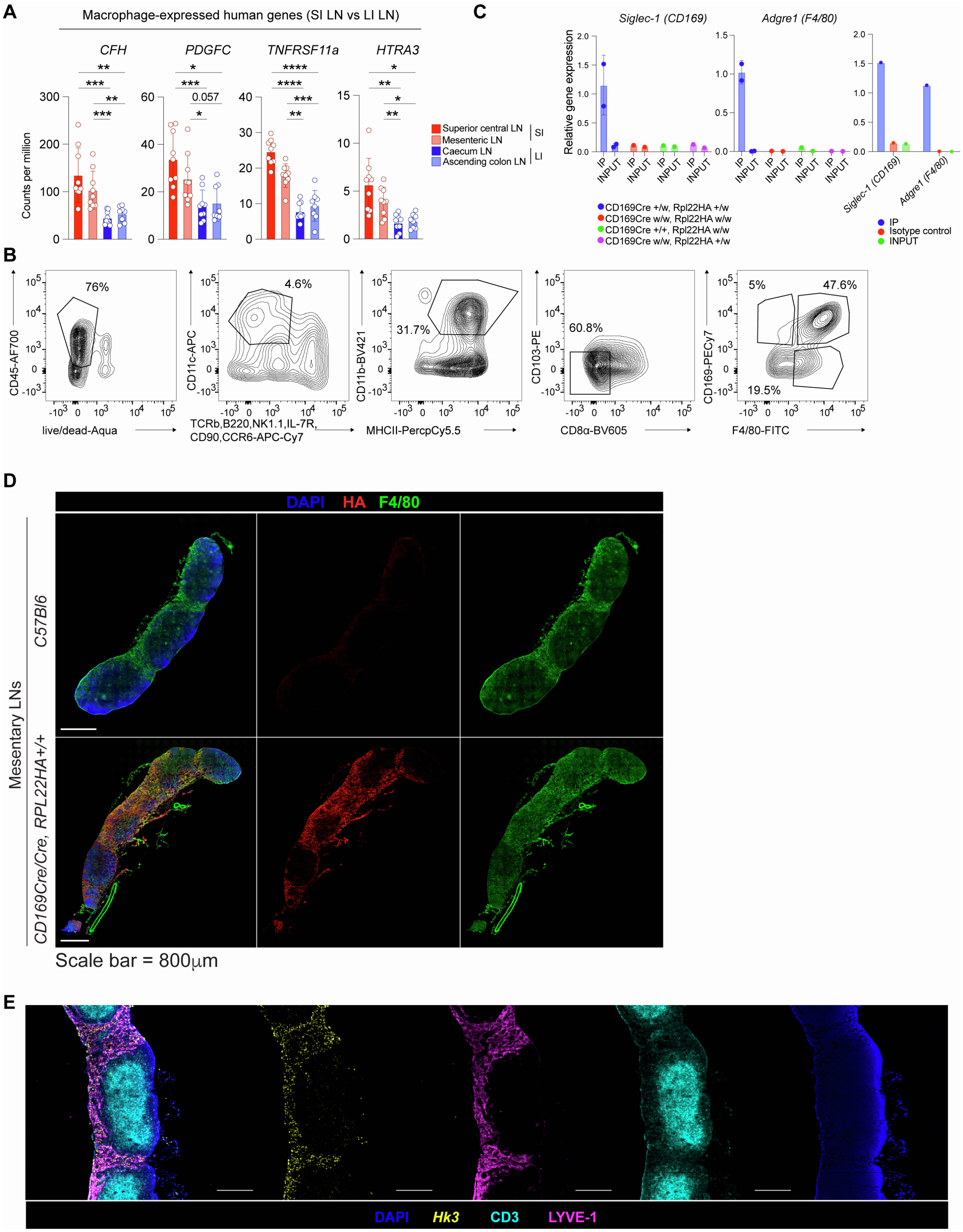

A
Macrophage-expressed human genes (SI LN vs LI LN)
CFH
PDGFC
TNFRSF11a
HTRA3
Counts per million
0.057
Superior central LN
Mesenteric LN
Caecum LN
Ascending colon LN
SI
LI
C
Siglec-1 (CD169)
Adgre1 (F4/80)
Relative gene expression
IP
INPUT
CD169Cre +/w, Rpl22HA +/w
CD169Cre w/w, Rpl22HA w/w
CD169Cre +/+, Rpl22HA w/w
CD169Cre w/w, Rpl22HA +/w
Isotype control
B
76%
4.6%
31.7%
60.8%
5%
47.6%
19.5%
CD45-AF700
live/dead-Aqua
CD11c-APC
TCRb,B220,NK1.1,IL-7R, CD90,CCR6-APC-Cy7
CD11b-BV421
MHCII-PercpCy5.5
CD103-PE
CD8α-BV605
CD169-PECy7
F4/80-FITC
D
DAPI HA F4/80
Mesentary LNs
C57Bl6
CD169Cre/Cre, RPL22HA+/+
Scale bar = 800μm
E
DAPI Hk3 CD3 LYVE-1

S5. Murine medullary sinus macrophages are more abundant in small intestinal compared to colonic LNs and transcriptionally distinct. A. Normalized counts per million of macrophage transcripts from indicated LNs as determined by *RNAseq*. Superior Central LN *n*=9, Mesenteric LN *n*=9, Caecum LN *n*=9, Ascending colon LN *n*=8. **B.** Gating strategy for LN macrophages. **C**. Fold enrichment of mRNA encoding for macrophage marker *Siglec1* and *Adgre* after RPL22-HA pulldown from CD169-Cre positive (+/w) *versus* negative (w/w) mice using an anti-HA antibody (left) or in CD169-Cre +/w mice using an anti-HA *versus* isotype control antibody for the pulldown, as assessed by QPCR. **D.** Representative sections of SI LNs from C57BL/6 or CD169-Cre RPL22HA mice stained with DAPI and anti-HA (green), LYVE-1 (red) or F4/80 (magenta). Bar= 400 μm. **E**. Representative sections of SI LNs from C57BL/6 mice stained immunofluorescently with DAPI (blue), an RNA probe against *Hk3* (yellow), anti-CD3 (cyan) or LYVE-1 (magenta). Bar = 400 μm. Data are represented as mean +/- SEM, * $p<0.05$, ** $p<0.01$, *** $p<0.001$, **** $p<0.0001$ by paired t-test.

Figure S6

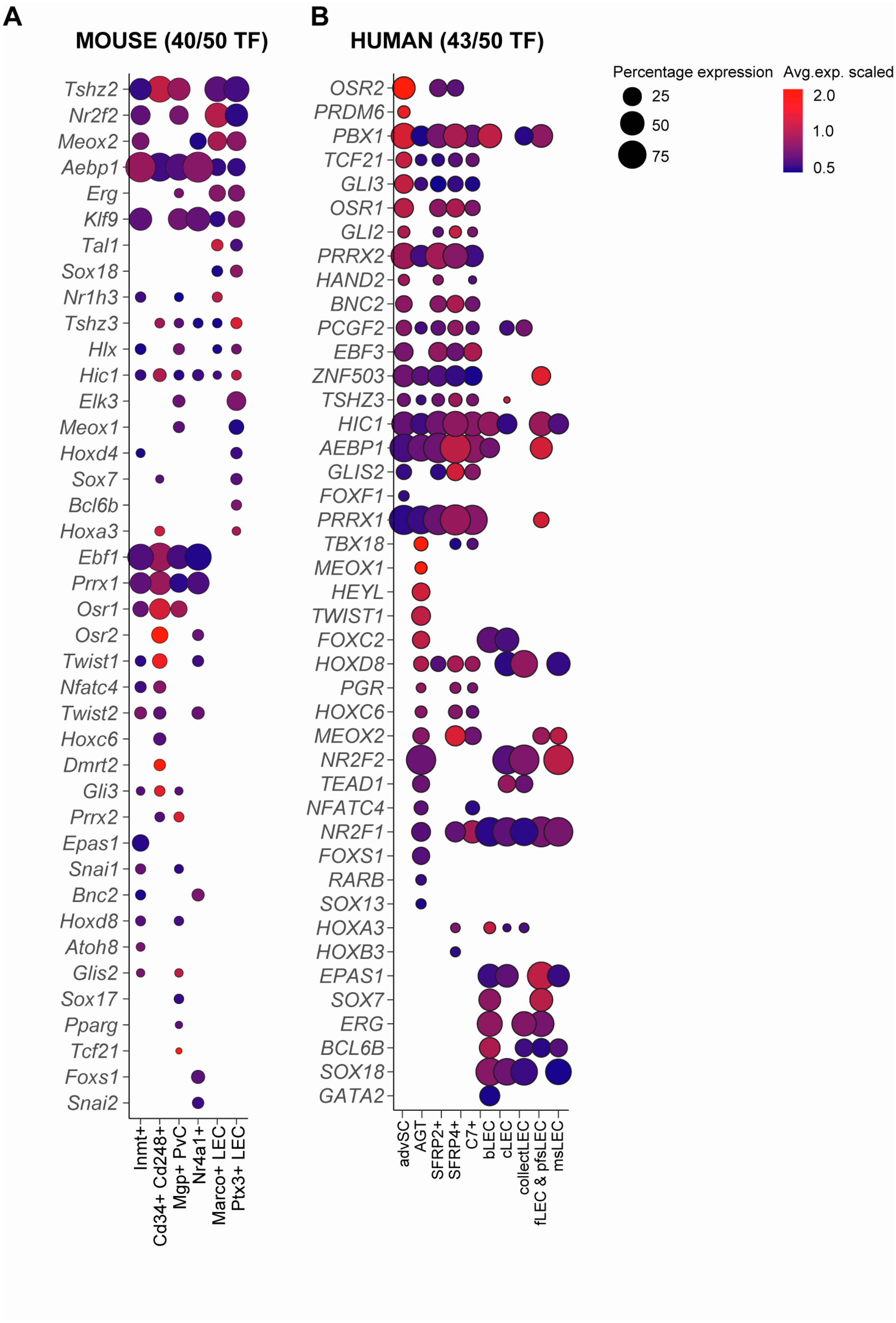
A
MOUSE (40/50 TF)
Tshz2
Nr2f2
Meox2
Aebp1
Erg
Klf9
Tal1
Sox18
Nr1h3
Tshz3
Hlx
Hic1
Elk3
Meox1
Hoxd4
Sox7
Bcl6b
Hoxa3
Ebf1
Prrx1
Osr1
Osr2
Twist1
Nfatc4
Twist2
Hoxc6
Dmrt2
Gli3
Prrx2
Epas1
Snai1
Bnc2
Hoxd8
Atoh8
Glis2
Sox17
Pparg
Tcf21
Foxs1
Snai2
Inmt+
Cd34+ Cd248+
Mgp+ PvC
Nr4a1+
Marco+ LEC
Ptx3+ LEC
B
HUMAN (43/50 TF)
OSR2
PRDM6
PBX1
TCF21
GLI3
OSR1
GLI2
PRRX2
HAND2
BNC2
PCGF2
EBF3
ZNF503
TSHZ3
HIC1
AEBP1
GLIS2
FOXF1
PRRX1
TBX18
MEOX1
HEYL
TWIST1
FOXC2
HOXD8
PGR
HOXC6
MEOX2
NR2F2
TEAD1
NFATC4
NR2F1
FOXS1
RARB
SOX13
HOXA3
HOXB3
EPAS1
SOX7
ERG
BCL6B
SOX18
GATA2
advSC
AGT
SFRP2+
SFRP4+
C7+
bLEC
cLEC
collectLEC
fLEC & pfsLEC
msLEC
Percentage expression
25
50
75
Avg.exp. scaled
2.0
1.0
0.5

S6. Small intestinal LN enriched cellular and transcriptional signatures are conserved between mouse and human. A, **B**. Dot plot of relative and percentage expression of transcription factors predicted to regulate DEGs between SI and LI LNs of mice (A) or humans (B) in indicated stromal cell populations.

Figure S7

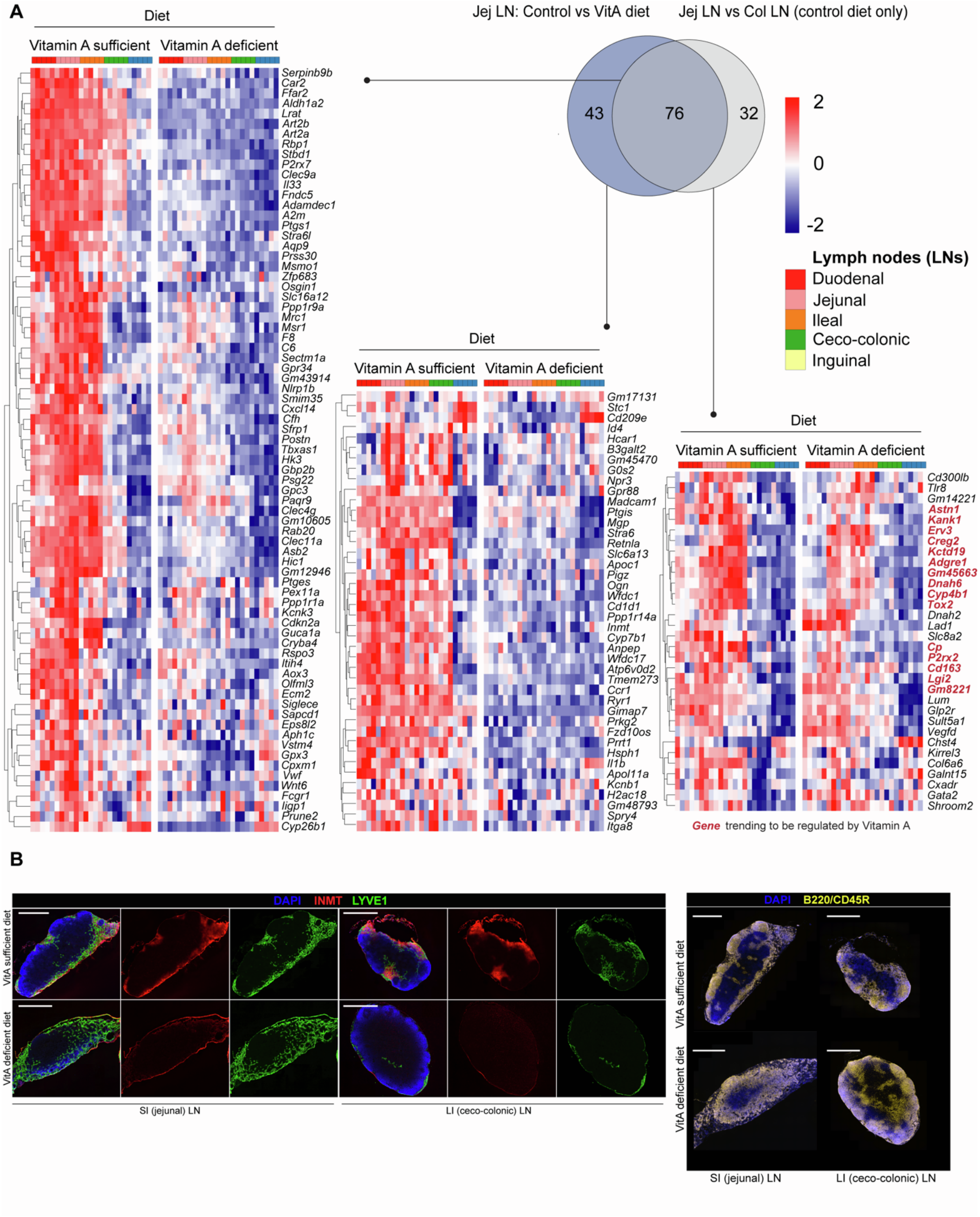
A
Diet
Vitamin A sufficient
Vitamin A deficient
Serpinb9b
Car2
Ffar2
Aldh1a2
Lrat
Art2b
Art2a
Rbp1
Stbd1
P2rx7
Clec9a
Il33
Fndc5
Adamdec1
A2m
Ptgs1
Stra6l
Aqp9
Prss30
Msmo1
Zfp683
Osgin1
Slc16a12
Ppp1r9a
Mrc1
Msr1
F8
C6
Sectm1a
Gpr34
Gm43914
Nlrp1b
Smim35
Cxcl14
Cfh
Sfrp1
Postn
Tbxas1
Hk3
Gbp2b
Psg22
Gpc3
Paqr9
Clec4g
Gm10605
Rab20
Clec11a
Asb2
Hic1
Gm12946
Ptges
Pex11a
Ppp1r1a
Kcnk3
Cdkn2a
Guca1a
Cryba4
Rspo3
Itih4
Aox3
Olfml3
Ecm2
Siglece
Sapcd1
Eps8l2
Aph1c
Vstm4
Gpx3
Cpxm1
Vwf
Wnt6
Fcgr1
Iigp1
Prune2
Cyp26b1
Jej LN: Control vs VitA diet
Jej LN vs Col LN (control diet only)
43
76
32
2
0
-2
Lymph nodes (LNs)
Duodenal
Jejunal
Ileal
Ceco-colonic
Inguinal
Diet
Vitamin A sufficient
Vitamin A deficient
Gm17131
Stc1
Cd209e
Id4
Hcar1
B3galt2
Gm45470
G0s2
Npr3
Gpr88
Madcam1
Ptgis
Mgp
Stra6
Retnla
Slc6a13
Apoc1
Pigz
Ogn
Wfdc1
Cd1d1
Ppp1r14a
Inmt
Cyp7b1
Anpep
Wfdc17
Atp6v0d2
Tmem273
Ccr1
Ryr1
Gimap7
Prkg2
Fzd10os
Prrt1
Hsph1
Il1b
Apol11a
Kcnb1
H2ac18
Gm48793
Spry4
Itga8
Diet
Vitamin A sufficient
Vitamin A deficient
Cd300lb
Tlr8
Gm14221
Astn1
Kank1
Erv3
Creg2
Kctd19
Adgre1
Gm45663
Dnah6
Cyp4b1
Tox2
Dnah2
Lad1
Slc8a2
Cp
P2rx2
Cd163
Lgi2
Gm8221
Lum
Glp2r
Sult5a1
Vegfd
Chst4
Kirrel3
Col6a6
Galnt15
Cxadr
Gata2
Shroom2
Gene trending to be regulated by Vitamin A
B
DAPI INMT LYVE1
VitA sufficient diet
VitA deficient diet
SI (jejunal) LN
LI (ceco-colonic) LN
DAPI B220/CD45R
VitA sufficient diet
VitA deficient diet
SI (jejunal) LN
LI (ceco-colonic) LN

S7. Differences between murine LNs along the gut manifest at weaning age irrespective of the microbiota but in dependence on dietary Vitamin A. A. Venn diagram depicting overlap of number of DEGs between jejunal (jej) LNs from mice fed a Vitamin A sufficient *versus* deficient diet and jejunal *versus* ceco-colonic (col) LNs of mice fed a Vitamin A sufficient diet, and heat maps and hierarchal clustering of DEGs contributing to each part of the Venn diagram. Symbols of genes categorized as exclusively regulated by LN location and independent of diet that are trending to be regulated by Vitamin A are in red. **B**. Representative cross-section of murine jejunal and ceco-colonic LN from 6-week-old C57BL/6 mice fed a Vitamin A sufficient or deficient diet and stained immunofluorescently with DAPI (dark blue), anti-LYVE-1 (cyan), and anti-INMT (red) or, on a sequential section, with DAPI (dark blue) and anti-B220 (yellow). Bar=400 μm.
